# Mechanochemical vs Wet Approach for Directing CO_2_ Capture toward Various Carbonate and Bicarbonate Networks

**DOI:** 10.1021/acssuschemeng.1c08402

**Published:** 2022-04-01

**Authors:** Michał K. Leszczyński, Dawid Kornacki, Michał Terlecki, Iwona Justyniak, Goran I. Miletić, Ivan Halasz, Piotr Bernatowicz, Vadim Szejko, Janusz Lewiński

**Affiliations:** † Faculty of Chemistry, Warsaw University of Technology, Noakowskiego 3, 00-664 Warsaw, Poland; ‡ Institute of Physical Chemistry, Polish Academy of Sciences, Kasprzaka 44/52, 01-224 Warsaw, Poland; § Ruđ̵er Bošković Institute, Bijenička 54, 10000 Zagreb, Croatia

**Keywords:** Mechanochemistry, Liquid-assisted grinding, CO_2_ capture, Biguanide, Carbonate, Bicarbonate, Hydrogen-bonded networks

## Abstract

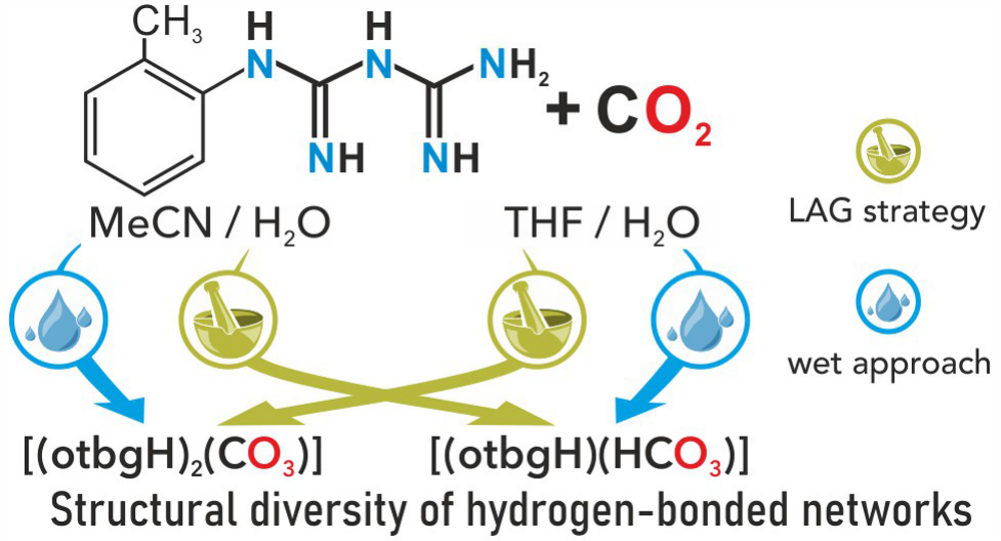

The distinct research areas related to CO_2_ capture and mechanochemistry are both highly attractive in the context of green chemistry. However, merger of these two areas, *i.e.*, mechanochemical CO_2_ capture, is still in an early stage of development. Here, the application of biguanidine as an active species for CO_2_ capture is investigated using both solution-based and liquid-assisted mechanochemical approaches, which lead to a variety of biguanidinium carbonate and bicarbonate hydrogen-bonded networks. We demonstrate that in solution, the formation of the carbonate vs bicarbonate networks can be directed by the organic solvent, while, remarkably, in the liquid-assisted mechanochemical synthesis employing the same solvents as additives, the selectivity in network formation is inversed. In general, our findings support the view of mechanochemistry not only as a sustainable alternative but rather as a complementary strategy to solution-based synthesis.

## Abstract 2

Mechanochemical vs solution-based approach to CO_2_ capture by nitrogen-rich organic bases leads to various hydrogen-bonded carbonate and bicarbonate biguanidinium networks.

## Introduction

Continuously increasing environmental awareness in the recent decades has encouraged the development of many sustainability-driven initiatives, defined within the Twelve Principles of Green Chemistry, aiming at enhanced efficiency and environmental safety of chemical processes.^1,2^ In this notion, the mechanically induced solid state chemical transformations have been elevated from mere curiosity to a dynamically growing research field providing highly efficient alternatives to the classic solution-based synthetic methods^3,4^ Within this recent advent of mechanochemistry, a broad range of remarkable developments have been demonstrated in synthetic organic^5−8^ and inorganic chemistry,^9−11^ as well as materials science.^12−18^ One of the emerging research fields in this area concerns mechanically induced chemical reactions at the gas–solid interface.^19^ With regard to the principles of green chemistry development of gas–solid-state small-molecule ball-milling processes involving CO_2_ would be highly desirable, but this research field is still at its infancy with only a handful of reports in the past decade. For example, Pinhas and co-workers used gaseous CO_2_ in the solvent-free and catalyst-free conversion of an aziridine to an oxazolidinone,^20^ and Métro and co-workers developed facile formation of l-lysine ammonium carbamate upon grinding of l-lysine in a CO_2_ atmosphere.^21^ Another interesting contribution concerned the graphite functionalization and exfoliation by ball milling in the presence of dry ice^22,23^ and probing the reactivity of a ZIF material toward gaseous CO_2_.^24^

**Scheme 1:**
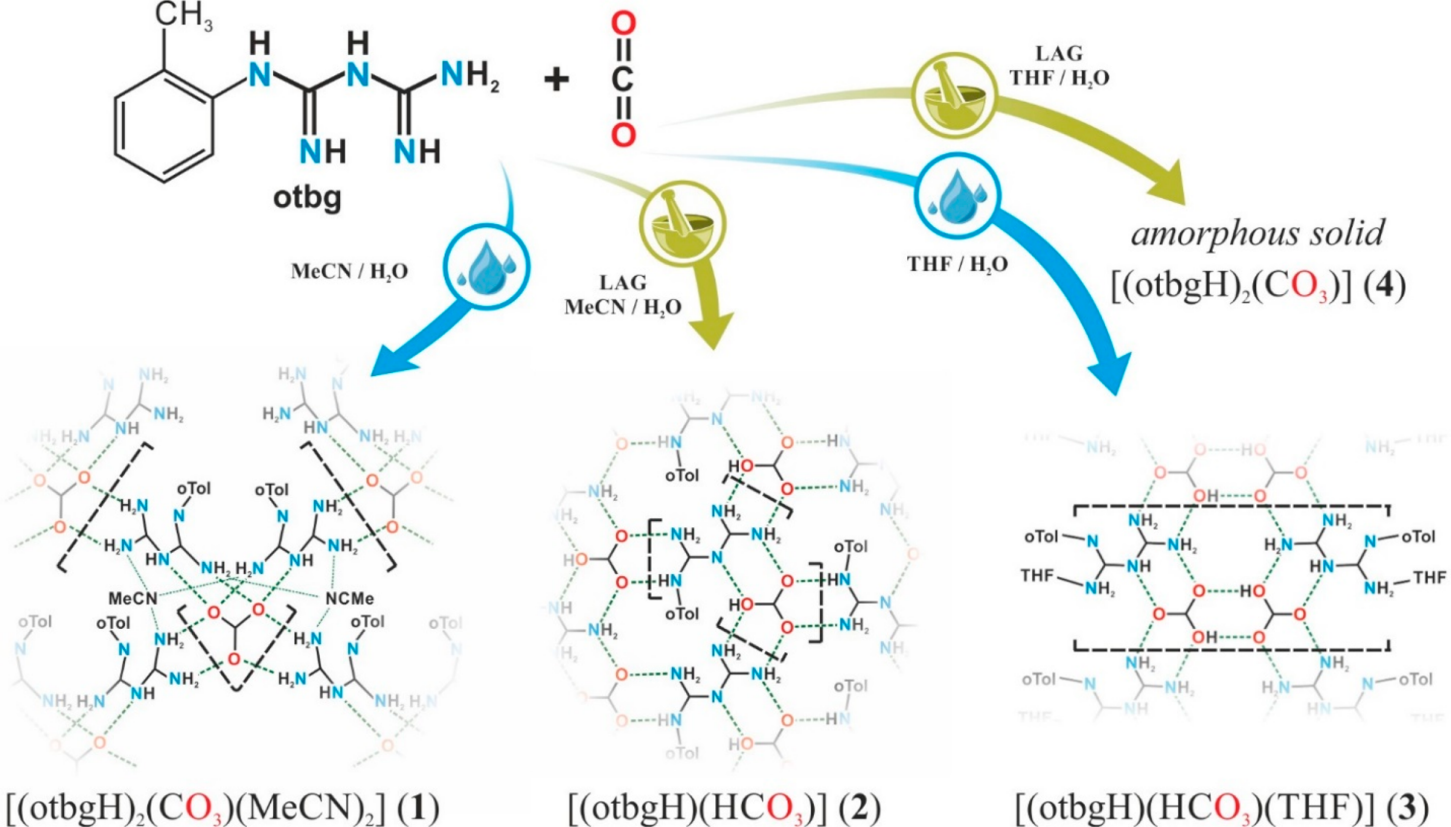
Reaction Pathways Leading to **1**–**4** Representing CO_2_ Fixation by otbg Using MeCN and THF in Solution-Based and LAG Strategies

Following the pioneering studies concerning CO_2_ fixation and conversion using nitrogen-rich organic bases,^25,26^ the metal-free approach aimed at CO_2_ conversion has evolved into one of the most promising green sources of C1 synthons over the past decade.^27−29^ In these mostly solution-based investigations, the initial reaction step involves formation of carbamate-type zwitterionic species, which can be hydrolyzed toward carbonates or bicarbonates. However, in-depth understanding of the mechanism of carbamate hydrolysis appears as a nontrivial task due to a number of possible reaction pathways including cooperative interactions of multiple molecules.^30^ In this regard, apart from the strongly basic amidine or guanidine derivatives, one of the most commonly studied CO_2_ absorption systems involves polymeric species functionalized with amine moieties.^31,32^ Therefore, a promising development appears to be the use of biguanide derivatives, which combine both high basicity and capabilities of internal proton transfer, but this research area appears to be surprisingly underdeveloped, with only a handful of reports related to the study of CO_2_ sensing platforms based on the polyhexamethylene biguanide (PHMB) polymer.^33,34^ As part of our systematic studies on both designing of various reaction systems in wet and solid state reaction environments^35−37^ as well as the use of CO_2_ as a substrate in the preparation of functional materials,^38−41^ herein we confront the mechanochemical vs solution approach to CO_2_ fixation by a model biguanide. Our investigations reveal that a variety of carbonate and bicarbonate products differing in molecular structures and supramolecular architectures can be formed in the studied reaction system depending on both the solvent (MeCN vs THF) and the methodology (solution vs liquid-assisted grinding (LAG)) used (Scheme 1). In particular, we found that the selectivity of carbonate vs bicarbonate formation in the solution-based method is related to the type of used solvent. Remarkably, if the same solvent was used for a LAG process, the reaction selectivity toward carbonate vs bicarbonate was reversed and accompanied by an extraordinary difference in the resulting supramolecular architecture of the hydrogen-bonded network, in comparison to the wet approach. Finally, the process of CO_2_ release form the developed biguanidinium networks was studied, revealing low decomposition temperatures, which is desirable for the application as controlled CO_2_ capture-and-release systems.

## Results and Discussion

**Figure 1:**
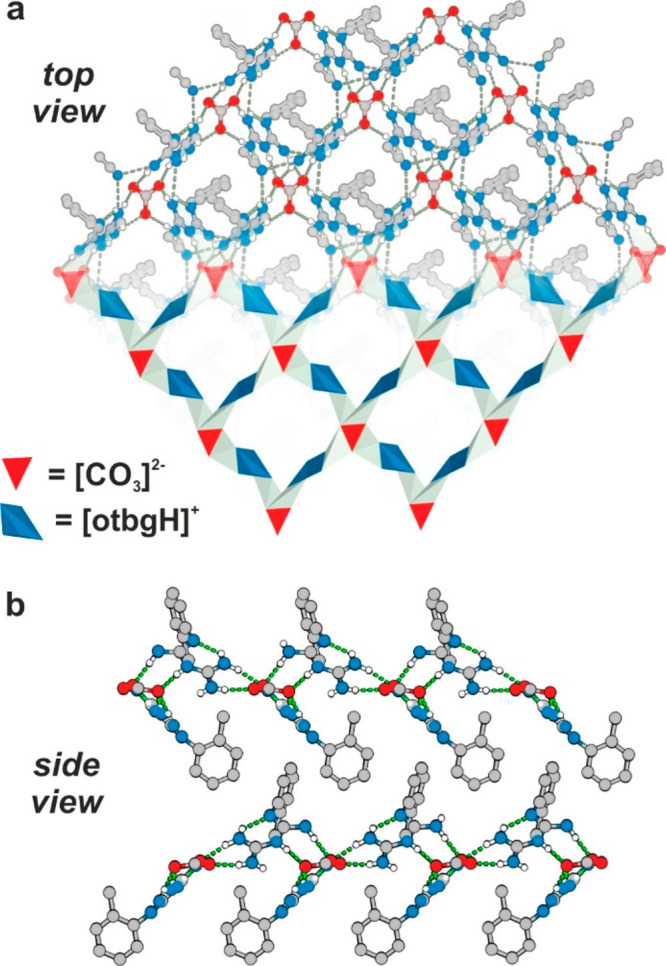
Crystal structure of **1**: (a) 2D supramolecular hydrogen-bonded network (o-tolyl groups have been omitted in picture for clarity). (b) Stacking of the 2D layers. C = gray, N = blue, O = red, H = white.

**Figure 2:**
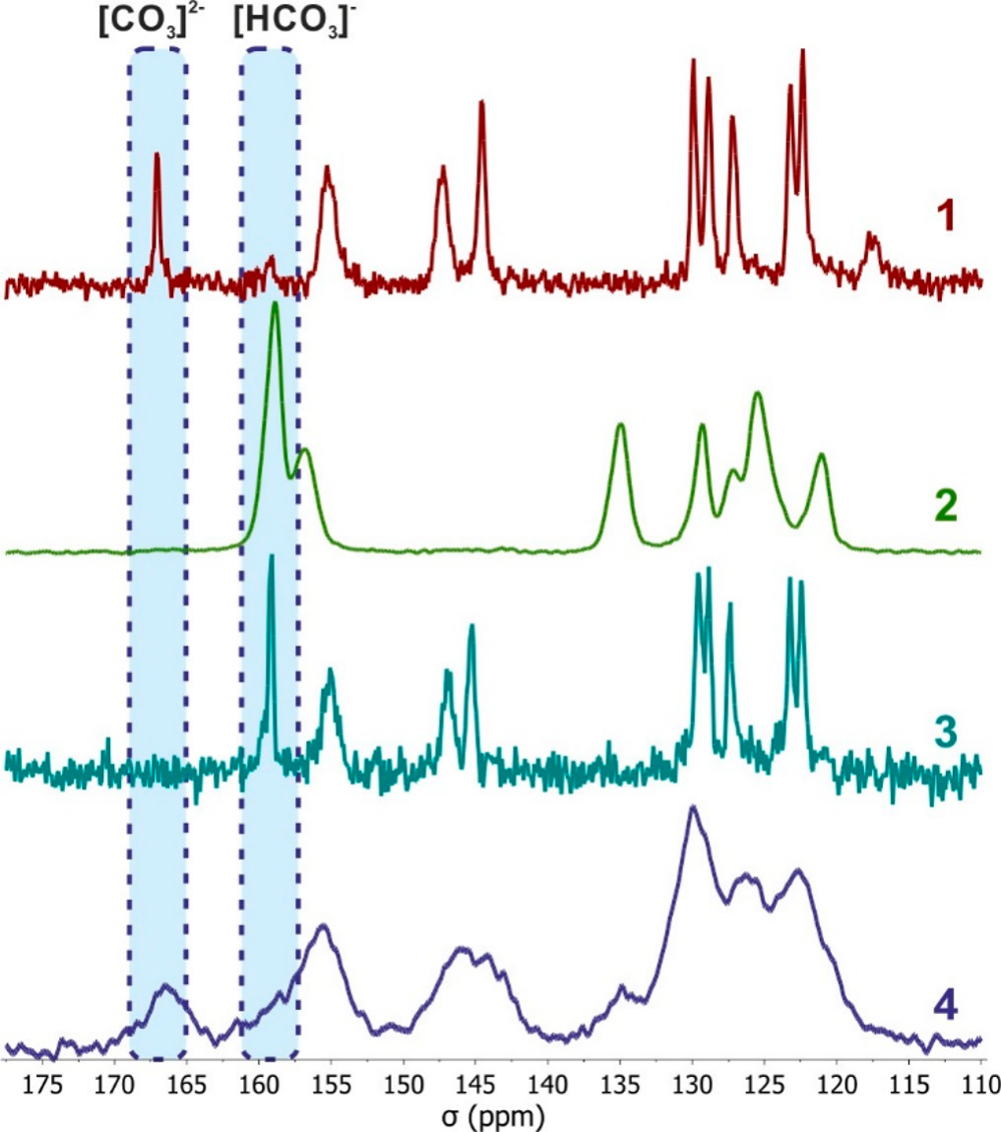
^13^C CP-MAS NMR spectra of **1**, **2**, **3**, and **4** (110–180 ppm range).

Initially, the CO_2_ capture process was studied in a MeCN/H_2_O solution environment. Exposing the MeCN/H_2_O (40:1 by volume) solution of 1-(o-tolyl)biguanide (otbg) to gaseous CO_2_ resulted in an immediate deposition of white precipitate, which was identified as a solvate [(otbgH)_2_(CO_3_)(MeCN)_2_] (**1**) using a variety of techniques (see below). High quality single crystals (*C*222_1_ space group) of **1** were prepared by slow diffusion of CO_2_ into a MeCN/H_2_O (40:1 vol) solution of otbg at room temperature after ca. 2 days. Single crystal X-ray diffraction studies (SCXRD) revealed that the crystal structure **1** consists of [otbgH]^+^ cations and carbonate dianions interconnected by hydrogen bonds, which results in the formation of a 2D supramolecular hydrogen-bonded network layers (Figure 1a; Figures S1 and S2) forming a 3D stacked structure (Figure 1b). Moreover, the crystal structure of **1** includes MeCN molecules, and each MeCN molecule bridges three independent biguanidinium moieties by hydrogen-bonded interactions (Scheme 1, bottom left corner) (N–N distances: 3.105(4), 3.154(3), and 3.169(4) Å). Significantly, **1** could also be easily prepared as a crystalline precipitate in good yield by direct air capture (DAC) of atmospherical CO_2_ upon exposure of a MeCN/H_2_O (40:1 vol) otbg solution to open air at −24 °C for 1 day. The identity and phase-purity of the prepared materials were confirmed using elemental analysis, PXRD (Figure S8), FTIR, and NMR spectroscopies. The FTIR spectrum of **1** (Figure S29) was rather complex but involved a signal at 1371 cm^–1^, related to the stretching mode of the carbonate anion. Moreover, the ^13^C CPMAS NMR spectrum (Figure 2; Figure S11) appeared very informative due to a characteristic sharp signal at 169 ppm, typical for carbonate ions. Purity of **1** was additionally confirmed using ^1^H and ^13^C NMR spectroscopies in D_2_O solution (Figures S15 and S16). Noteworthy, the materials capable of absorbing CO_2_ directly from air (at ca. 400 ppm concentration), such as iminoguanidinate-based systems,^42−45^ are highly desirable with regard to their use toward achieving the “negative CO_2_ emission” goal.

**Figure 3:**
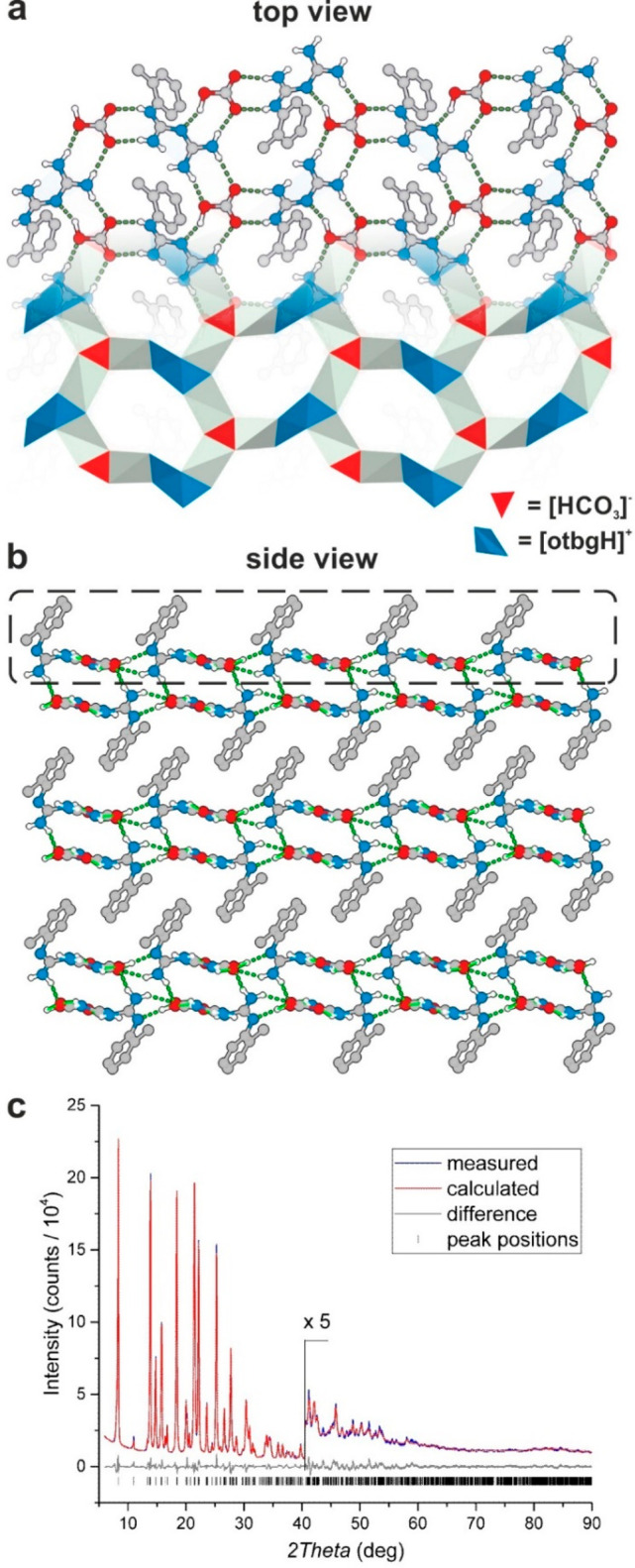
Crystal structure of **2**: (a) 2D supramolecular hydrogen-bonded network (half-layer), (b) stacking of the 2D layers (half-layer marked in rectangle), (c) PXRD diffractogram of **2** including experimental and fitted data. C = gray, N = blue, O = red, H = white.

Encouraged by the observation of extended hydrogen-bonded networks in **1**, we wondered if applying the mechanochemical approach could influence the reaction pathway and lead to formation of different products. Therefore, we conducted a mechanochemical reaction by grinding otbg with a stoichiometric amount of H_2_O (1:1) as well as a small amount (20 μL for 200 mg of the reaction mixture) of acetonitrile as an additive in a CO_2_-rich atmosphere, afforded by introduction of an excess of solid CO_2_ into the reaction vessel prior to grinding. As a result of the LAG process (15 min, 30 Hz), a white powder of a bicarbonate [(otbgH)(HCO_3_)] (**2**) was formed in almost quantitative yield. Numerous attempts at recrystallization of **2** were unsuccessful, but the crystal structure was solved by simulated annealing using PXRD data (Figure 3c; Figures S5 and S6). The PXRD data analysis revealed that the crystal structure of **2** is composed of biguanidinium cations and bicarbonate anions interconnected into an extended hydrogen-bonded network (Figure 3a and b; Figures S6 and S7). The exact location of protons in the biguanidinium moiety was additionally confirmed using DFT calculations (see Supporting Information for more details). Interestingly, the bicarbonate anions in **2** did not assemble into the characteristic dimeric moieties^46,47^ but formed monomers stabilized by seven hydrogen bonds (one donating and six accepting) formed with the surrounding four biguanidinium cations. The extended hydrogen-bonded network formed double-layered 2D sheets with 86% of the hydrogen bonds located within each layer and 14% devoted to linking the separate layers into the double-layered assembly, which, as a whole, formed a stacked 3D crystal structure (Figure 3b). Additionally, elemental analysis confirmed that **2** was prepared as a phase-pure solid (see Supporting Information for details), while both the FTIR spectrum with a signal at 1346 cm^–1^ (Figure S30) and the ^13^C CPMAS NMR spectrum with a signal at 161 ppm (Figure 2; Figure S12) well substantiated the presence of the bicarbonate ion. Additionally, ^1^H and ^13^C NMR spectra in D_2_O solution confirmed the purity of the product **2** (Figures S17 and S18).

**Figure 4:**
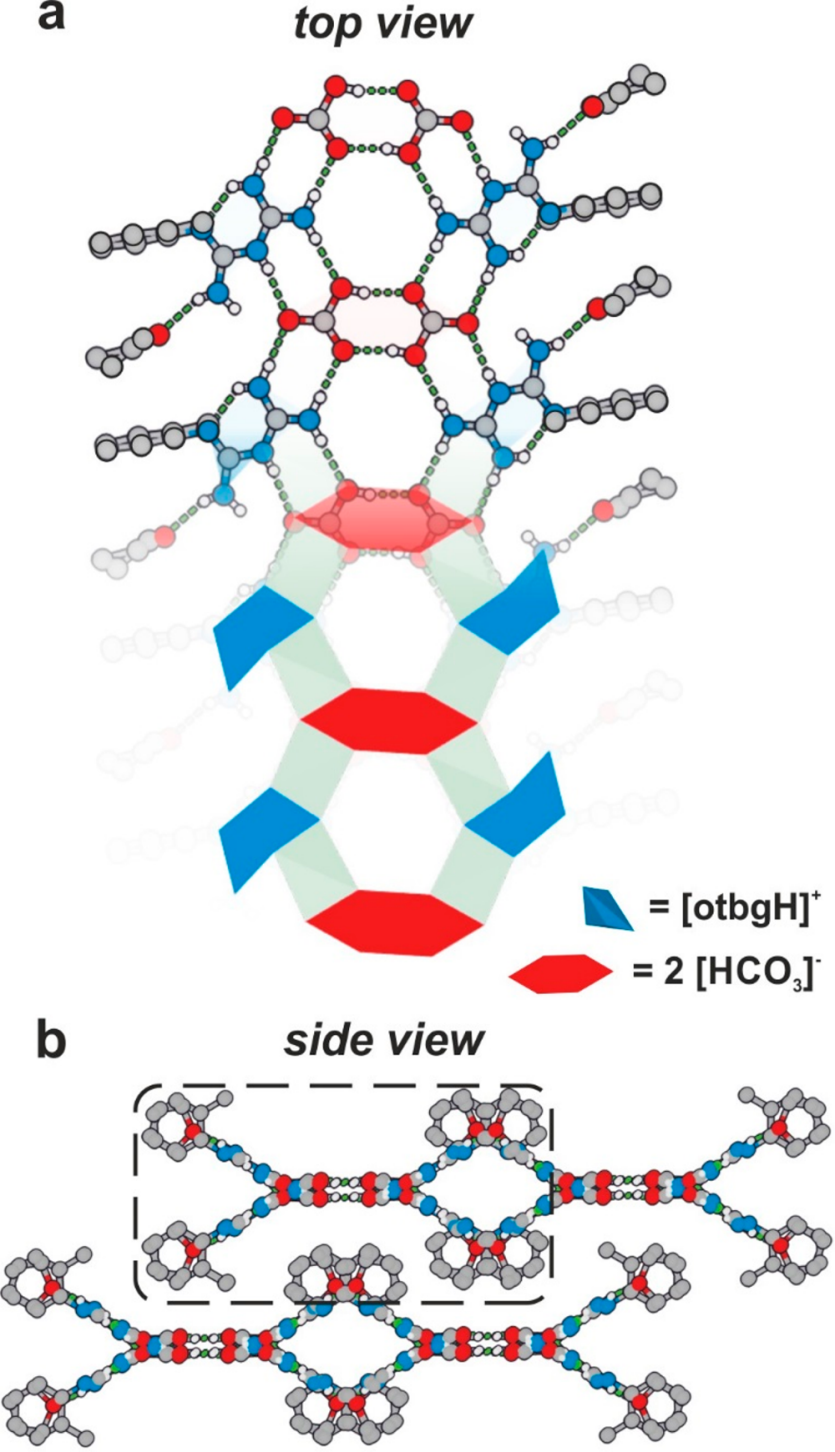
Crystal structure of **3**: (a) 1D supramolecular hydrogen-bonded ribbon, (b) stacking of the 1D ribbons in **3** (single ribbon marked in rectangle). C = gray, N = blue, O = red, H = white.

Having observed the acetonitrile molecules bridging the three independent biguanidinium moieties by hydrogen bonds in **1**, we wondered if using a different donor solvent could affect the structure of the formed network. Therefore, in next experiment, we used THF for the study of CO_2_ capture by otbg. Absorption of gaseous CO_2_ by a THF/H_2_O solution (50:1 by volume) of otbg resulted in precipitation of a solvated bicarbonate [(otbgH)(HCO_3_)(THF)] (**3**) in almost quantitative yield. Single crystals of **3** were prepared by slow diffusion of CO_2_ into a THF/H_2_O solution (50:1 by volume) of otbg. The identity and purity of **3** was confirmed using a variety of analytical techniques (see below). Analysis of the SCXRD data revealed that the crystal structure of **3** consists of bicarbonate dimers interconnected by biguanidinium cations (Figure 4a; Figures S3 and S4), which self-organized into an extended hydrogen-bonded network of a rosette-ribbon structure (Figure 4). The crystal structure of **3** does involve also THF solvent molecules coordinated by the N–H···O hydrogen bond to the biguanidinium moiety backbone (N–O distance of 2.827(3) Å) (Figure 4). The PXRD study (Figure S9) and elemental analysis confirmed that **3** was a phase-pure compound, and both the FTIR spectrum featuring a signal at 1343 cm^–1^ (Figure S31) and the ^13^C CPMAS NMR spectrum exhibiting a sharp signal at 161 ppm (Figure 2; Figure S13), as well as ^1^H and ^13^C NMR spectra in D_2_O solution (Figures S19 and S20), substantiated the presence of the bicarbonate species.

Additionally, the LAG procedure was involved to investigate the mechanochemical CO_2_ capture by otbg in the presence of THF and water. As a result, [(otbgH)_2_(CO_3_)] (**4**) was prepared as a white amorphous powder, as evidenced by a PXRD study (Figure S10), which hindered our attempts to investigate its internal structure. Despite our unsuccessful attempts to crystallize **4**, its chemical composition was studied using ^13^C CPMAS NMR (Figure 2; Figure S14), which revealed a broad signal at 168 ppm, characteristic for the CO_3_^2–^ species. The composition of **4** was additionally studied using FTIR spectroscopy, revealing a signal at 1367 cm^–1^ related to carbonate ions (Figure S32) and elemental analysis (see Supporting Information for details), which unequivocally confirmed the formula [(otbgH)_2_(CO_3_)]. Purity of **4** was additionally confirmed by analysis of the ^1^H and ^13^C NMR spectra in D_2_O solution (Figures S21 and S22).

As demonstrated, otbg appears as a promising molecule for CO_2_ capture applications. Despite the fact that absorption of CO_2_ using the typical amine-based systems (e.g., polyethylenimine) usually leads to formation of carbamate or carbamic acid derivatives,^48,49^ neither of these species were detected as products in conducted reactions involving otbg. Instead, all of the products were identified as pure carbonates or bicarbonates, which was confirmed by both solid state ^13^C CPMAS NMR (Figures 2; Figures S11–S14) and ^1^H and ^13^C NMR studies in D_2_O solutions of **1**, **2**, **3**, and **4** (Figures S15–S22), as well as elemental analysis. Thus, the observed results of CO_2_ absorption using otbg solutions are consistent with the literature reports concerning similar transformations involving guanidines, amidines, and urea, which form carbonates or bicarbonates upon exposure to CO_2_ in the presence of water.^25,26,50^ Nevertheless, the most desired properties of potential CO_2_ scrubbing systems concern not only capture but also easy and on-demand release of CO_2_. In this regard, we investigated the thermal decomposition of **1**, **2**, **3**, and **4** using thermogravimetric analysis (TGA), which revealed that in all of the studied cases CO_2_ can be released at low temperatures (Figures S24–S27). The thermal decomposition of all of the studied materials resulted in formation of otbg, as evidenced by the observed weight loss values corresponding to the release of CO_2_, H_2_O, and (optionally) organic solvent. The decomposition step leading to the formation of otbg was observed at relatively low temperatures: 89, 110, 93, and 115 °C for **1**, **2**, **3**, and **4**, respectively. Further thermal decomposition of the studied samples closely matched the decomposition pathway of the pure otbg sample, which additionally confirmed the proposed formation of the otbg phase in the initial decomposition step (Figure S28). Finally, we have studied the possibility for recovery of the otbg base by thermal decomposition (at 100 °C) of **1** and **3** followed by ^1^H NMR spectroscopic analysis (Figure S23). As a result, we found that **1** could be readily decomposed to obtg with essentially no signs of biguanide degradation even after five cycles of a CO_2_ absorption–thermal decomposition process (final yield of the collected otbg was 85% with respect to the starting material used in the initial cycle). In the case of **3**, the initial decomposition also allowed for recovery of the otbg, but extended repetition of this procedure resulted in partial degradation of otbg (Figure S23).

## Conclusions

In conclusion, we demonstrated that the CO_2_ capture process using the studied biguanide system in the presence of water and organic solvents leads to various hydrogen-bonded structures depending on the solvent type and application of solution-based or mechanochemical strategy. In particular, we found that the controlled formation of carbonate vs bicarbonate networks could be achieved by selection of the organic solvent. Remarkably, the reaction selectivity toward carbonate/bicarbonate is inversed if the mechanochemical approach is used instead, which was also accompanied by a substantial change in the supramolecular architecture of the resulting hydrogen-bonded networks. Moreover, thermal CO_2_ release from the studied materials was investigated, revealing low temperatures of thermal decomposition, which is desirable for controlled CO_2_ capture-and-release applications. In general, our findings support the view of mechanochemistry not only as a sustainable alternative but also as a complementary strategy to solution synthesis, which might lead to products unavailable using the traditional wet-chemistry procedures. Further investigations in this research area, including application of a range of other organic derivatives and various CO_2_ capture conditions, are currently underway.

## Supporting Information Available

Experimental procedures, supporting figures and tables concerning single crystal X-ray diffraction, powder X-ray diffraction, NMR spectroscopy, TGA, and IR spectroscopy (PDF)
Crystallographic data for product **1** (CCDC 2099296) based on the single crystal X-ray diffraction study (CIF)
Crystallographic data for product **2** (CCDC 2093967) based on the powder diffraction study (CIF)
Crystallographic data for product **3** (CCDC 2099297) based on the single crystal X-ray diffraction study (CIF)

## Supplementary Material

sc1c08402_si_001.pdf

sc1c08402_si_002.cif

sc1c08402_si_003.cif

sc1c08402_si_004.cif
