# Supplementary material for: Mechanochemical vs Wet Approach for Directing CO2 Capture toward Various Carbonate and Bicarbonate Networks
Source: ACS Sustain Chem Eng. 2022 Apr 1;10(14):4374–80. doi: 10.1021/acssuschemeng.1c08402 (PMC9006257; doi:10.1021/acssuschemeng.1c08402)
Supplement: Supplementary file 1 — sc1c08402_si_001.pdf [file sc1c08402_si_001.pdf]

Supporting information

Mechanochemical vs Wet Approach for Directing CO_2 capture toward Various Carbonate and Bicarbonate Networks

Michał K. Leszczyński,[a,b] Dawid Kornacki,[b] Michał Terlecki,[a] Iwona Justyniak,[b] Goran I. Miletić,[c] Ivan Halasz,[c] Piotr Bernatowicz,[b] Vadim Szejko,[a] and Janusz Lewiński*[a,b]

[a] Warsaw University of Technology, Faculty of Chemistry, Noakowskiego 3, 00-664 Warsaw, Poland
[b] Polish Academy of Sciences, Institute of Physical Chemistry, Kasprzaka 44/52, 01-224 Warsaw, Poland
[c] Ruđer Bošković Institute, Bijenička 54, 10000 Zagreb, Croatia

Number of Pages: 27

Number of Figures: 32

Number of Tables: 2

Table of contents:

1. Experimental section S3
2. Single crystal X-ray diffraction study S5
3. Powder X-ray diffraction study S11
4. NMR study S16
5. Thermogravimetric analysis S23
6. IR spectroscopy S26
7. References S28

1. Experimental section

General considerations

All chemicals were purchased from commercial sources and used without further purification. Gaseous CO_2 (99.995%) was purchased and stored in steel pressurised container and dosed using rubber balloon. Solid CO_2 was prepared by sublimation of the gaseous CO_2 using liquid nitrogen cooled glass apparatus. All of the operations were conducted in glass vessels unless otherwise noted. All of the mechanochemical reactions were conducted in stainless steel reactor (5 ml) equipped with three stainless steel balls (7 mm in diameter). Elemental analysis experiments were carried out using an UNICUBE Elementar (GmbH) analyser.

Synthesis of [(otbgH)$_2$(CO$_3$)(MeCN)$_2$] (1). Solution of otbg (200 mg, 1.05 mmol) in 4 ml of MeCN and 0.1 ml of H_2O (5.6 mmol) was put in a 50 ml glass vessel. Next, 25 ml of pure gaseous CO_2 was gently added to the reaction vessel using syringe and the vessel was sealed. Immediately white precipitate was formed. After 48h the resulting suspension was centrifuged and the white solid was dried in air yielding **1** (220 mg, 79.9%). Elemental analysis (%) calcd. for [$C_{23}H_{34}N_{12}O_3$]: C 52.47, H 6.464, N 31.94; found: C 52.75, H 6.475, N 32.10. ^{1}H NMR (D_2O, 25˚C): δ 7.24 – 7.05 (C_{-arom}-H), 2.12 (CH_{3-otbg}), 1.93 (CH_{3-MeCN}); ^{13}C NMR (D_2O, 25˚C): δ 160.29 (CN_{3-otbg}), 158.55 (CO_3), 134.77 – 126.59 (C_{-arom}), 119.03 (CN_{-MeCN}), 16.64 (CH_{3-otbg}), 0.77 (CH_{3-MeCN}); ^{13}C NMR (CPMAS, solid state, 25˚C): 169.16 (CO_3), 156.97 (CN_3), 148.63 (CN_3), 145.88 (N-C_{arom}), 130.66 – 122.80 (C_{arom}), 16.69 (CH_3).

Synthesis of [(otbgH)(HCO$_3$)] (2). Reaction vessel was loaded with otbg (200 mg, 1.05 mmol), H_2O (19 µl, 1.05 mmol), MeCN (20 µl, 0.38 mmol) and solid CO_2 (70 mg, 1.59 mmol). Then the reaction vessel was closed and shaken for 15 min with a frequency of 30 Hz. Product **2** was obtained as a white powder. Yield: 260 mg (98%). Elemental analysis (%) calcd for [$C_{10}H_{15}N_5O_3$]: C 47.43, H 5.929, N 27.67; found: C 47.88, H 5.767, N 28.17. ^{1}H NMR (D_2O, 25˚C): δ 7.25 – 7.13 (C_{-arom}-H), 2.14 (CH_{3-otbg}); ^{13}C NMR (D_2O, 25˚C): δ 160.53 – 160.47 (CN_{3-otbg}), 158.72 (CO_3), 135.51 – 126.98 (C_{-arom}), 16.57 (CH_{3-otbg}); ^{13}C NMR (CPMAS, solid state, 25˚C): 160.72 (HCO_3), 158.50 (CN_3), 135.87 (CN_3), 130.02 – 121.45 (C_{arom}), 20.28 (CH_3).

Synthesis of [(otbgH)(HCO$_3$)(THF)] (3). Solution of otbg (100 mg, 0.52 mmol) in 5 ml of THF and 0.1 ml of H_2O (5.6 mmol) was prepared. Next, gaseous CO_2 was added to the solution by bubbling for 20 seconds. Compound **3** was obtained as white precipitate, which was isolated by centrifugation and dried for 48h at room temperature. Yield: 169 mg (99.3%). Elemental analysis (%) calcd for [$C_{14}H_{23}N_5O_4$]: C 51.68, H 7.13, N 21.52; found: C 52.25, H 7.093, N 21.72. ^{1}H NMR (D_2O, 25˚C): δ 7.26 – 7.13 (C_{-arom}-H), 3.63 – 3.60 (CH_{2-THF}), 2.14 (CH_{3-otbg}), 1.77 – 1.73 (CH_{2-THF}); ^{13}C NMR (D_2O, 25˚C): δ 160.48 – 160.36 (CN_{3-otbg}), 158.72 (CO_3), 135.53 – 126.98 (C_{-arom}), 67.76 (CH_{2-THF}), 24.93 (CH_{2-THF}), 16.57 (CH_{3-otbg}), 0.77 (CH_{3-MeCN}); ^{13}C NMR (CPMAS, solid state, 25˚C): 161.82 (HCO_3), 156.93 (CN_3), 146.78 (CN_3), 144.39 (N-C_{arom}), 130.53 – 121.03 (C_{arom}), 17.17 (CH_3).

Synthesis of [(otbgH)$_2$(CO$_3$)] (4). Reaction vessel was loaded with with otbg (200 mg, 1.05 mmol), H_2O (19 µl, 1.05 mmol), THF (20 µl, 0.24 mmol) and solid CO_2 (70 mg, 1.59 mmol). Then the reaction vessel was closed and shaken for 15 min with a frequency of 30 Hz. Product **2** was obtained as a white powder. Yield: 227 mg (97.6%). Elemental analysis (%) calcd for [$C_{19}H_{28}N_{10}O_3$]: C 51.35, H 6.306, N 31.53; found: C 51.95, H 6.522, N 30.77. ^{1}H NMR (D_2O, 25˚C): δ 7.24 – 7.05 (C_{-arom}-H), 2.12 (CH_{3-otbg}); ^{13}C NMR (D_2O, 25˚C): δ 160.27 (CN_{3-otbg}), 158.54 (CO_3), 136.47 – 126.52 (C_{-arom}),

16.64 ($CH_{3\text{-otbg}}$); ^{13}C NMR (CPMAS, solid state, 25˚C): 168.51 (CO_3), 157.33 (CN_3), 147.22 (CN_3), 136 – 123 (C_{arom}), 17.99 (CH_3).

Thermal decomposition procedure. Samples were placed in glass vials and put into an oven for 2h at 100 °C. Afterwards the samples were cooled to the room temperature and analysed.

2. Single crystal X-ray diffraction study

The crystals were selected under Paratone-N oil, mounted on the nylon loops and positioned in the cold stream on the diffractometer. The X-ray data for complexes **1** and **3** were collected at 100(2)K on a SuperNova Agilent diffractometer using graphite monochromated Cu$K\alpha$ radiation (λ = 1.54184 Å). The data were processed with *CrysAlisPro*.[1] The structures were solved by direct methods using the SHELXS-97 program and were refined by full matrix least–squares on F^2 using the program SHELXL.[2] All non-hydrogen atoms were refined with anisotropic displacement parameters. Hydrogen atoms were added to the structure model at geometrically idealized coordinates and refined as riding atoms. Crystallographic data (excluding structure factors) for the structure reported in this paper have been deposited with the Cambridge Crystallographic Data Centre as supplementary publication. Copies of the data can be obtained free of charge on application to CCDC, 12 Union Road, Cambridge CB21EZ, UK (fax: (+44)1223-336-033; e-mail: deposit@ccdc.cam.ac.uk). CCDC:

Crystal data and structure refinement for **1**	**(CCDC 2099296)**	
Moiety formula	$2(C_9H_{14}N_5)$, CO_3, $2(C_2H_3N)$	
Empirical formula	$C_{23}H_{34}N_{12}O_3$	
Formula weight	526.62	
Temperature	100(2) K	
Wavelength	1.54184 Å	
Crystal system	Orthorhombic	
Space group	C 2 2 21	
Unit cell dimensions	a = 11.5579(6) Å	a= 90°.
	b = 11.3670(3) Å	b= 90°.
	c = 21.6837(8) Å	g = 90°.
Volume	2848.8(2) $Å^3$	
Z	4	
Density (calculated)	1.228 Mg/m^3	
Absorption coefficient	0.713 mm^{-1}	
F(000)	1120	
Crystal size	0.24 x 0.18 x 0.11 mm^3	
Theta range for data collection	4.078 to 69.965°.	
Index ranges	-10<=h<=13, -7<=k<=13, -26<=l<=9	
Reflections collected	2722	
Independent reflections	1892 [R(int) = 0.0192]	
Completeness to theta = 67.684°	88.6 %	
Absorption correction	Semi-empirical from equivalents	
Max. and min. transmission	0.925 and 0.857	
Refinement method	Full-matrix least-squares on F^2	
Data / restraints / parameters	1892 / 0 / 175	
Goodness-of-fit on F^2	1.088	
Final R indices [I>2sigma(I)]	R1 = 0.0325, wR2 = 0.0775	

R indices (all data)	R1 = 0.0381, wR2 = 0.0799
Absolute structure parameter	0.1(2)
Extinction coefficient	n/a
Largest diff. peak and hole	0.144 and -0.158 e. Å^{-3}

Table S1. Hydrogen bonds for **1** [Å and °].

contact	d(H···A)	d(D···A)	<(D−H···A)	symmetry
N(5)-H(5B)...N(1)	2.02	2.675(3)	130.1	*x, y, z*
N(4)-H(4A)...O(2)	1.93	2.756(2)	156.9	*x, y, z*
N(5)-H(5A)...O(1)	1.97	2.812(3)	159.0	*x, y, z*
N(3)-H(3A)...O(1)	2.96	3.589(3)	129.5	*x-1/2, y-1/2, z*
N(2)-H(2B)...O(1)	2.00	2.829(3)	157.2	*x-1/2, y-1/2, z*

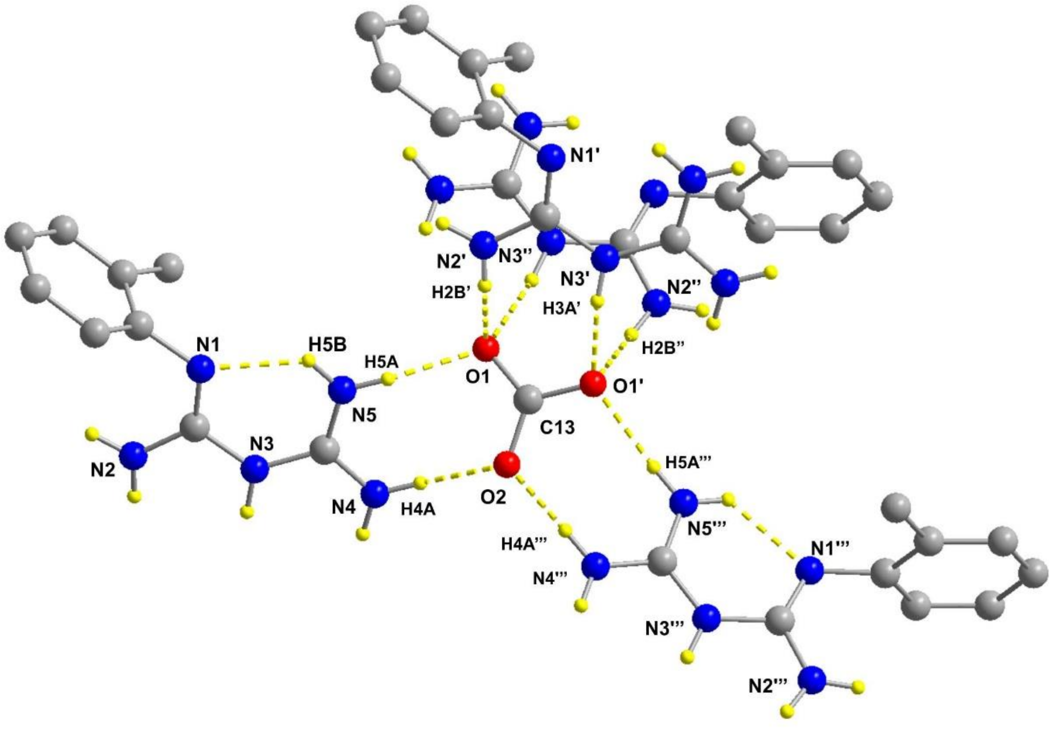

Figure S1. Hydrogen bonding in the structure of compound **1**. Hydrogen bonds are depicted with thick yellow dashed lines.

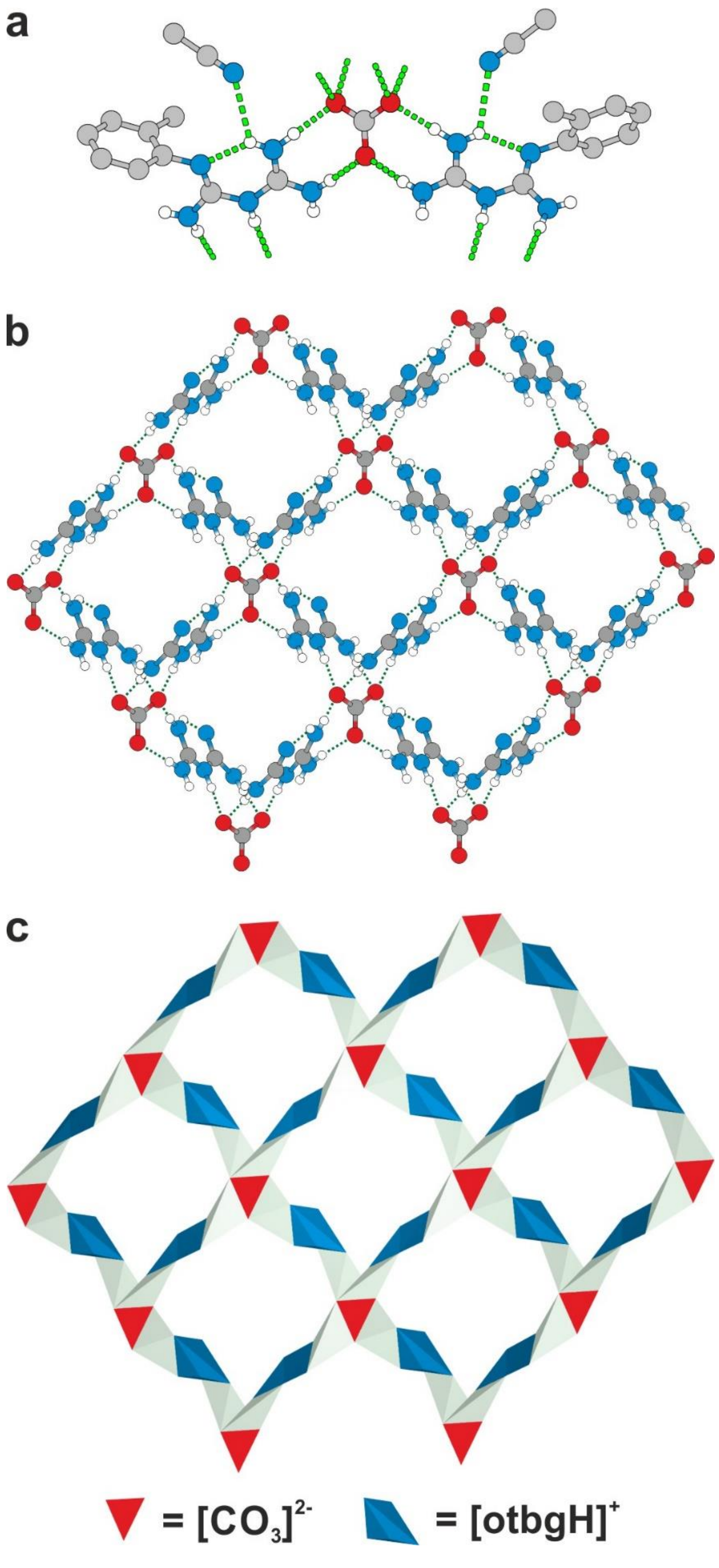

Figure S2. Crystal structure of **1** demonstrating the hydrogen-bonded network structure: a) simple building units, b) 2D supramolecular hydrogen-bonded network (MeCN molecules and o-tolyl groups have been omitted in picture for clarity), c) topological representation of the 2D hydrogen-bonded network C = grey, N = blue, O = red.

Crystal data and structure refinement for **3** **(CCDC 2099297)**

Moiety formula	$C_9H_{14}N_5$, C_4H_8O, CHO_3	
Empirical formula	$C_{14}H_{23}N_5O_4$	
Formula weight	325.37	
Temperature	100(2) K	
Wavelength	1.54184 Å	
Crystal system	Monoclinic	
Space group	C 2/c	
Unit cell dimensions	a = 18.226(3) Å	a= 90°.
	b = 16.0494(13) Å	b= 129.69(3)°.
	c = 15.145(2) Å	g = 90°.
Volume	3409.1(14) $Å^3$	
Z	8	
Density (calculated)	1.268 Mg/m^3	
Absorption coefficient	0.787 mm^{-1}	
F(000)	1392	
Crystal size	0.22 x 0.16 x 0.10 mm^3	
Theta range for data collection	4.079 to 71.369°.	
Index ranges	-14<=h<=22, -16<=k<=19, -18<=l<=18	
Reflections collected	6486	
Independent reflections	3250 [R(int) = 0.0338]	
Completeness to theta = 67.684°	99.7 %	
Absorption correction	Semi-empirical from equivalents	
Max. and min. transmission	0.924 and 0.860	
Refinement method	Full-matrix least-squares on F^2	
Data / restraints / parameters	3250 / 6 / 222	
Goodness-of-fit on F^2	1.070	
Final R indices [I>2sigma(I)]	R1 = 0.0571, wR2 = 0.1606	
R indices (all data)	R1 = 0.0658, wR2 = 0.1724	
Extinction coefficient	n/a	
Largest diff. peak and hole	0.438 and -0.265 e.$Å^{-3}$	

Table S2. Hydrogen bonds for **3** [Å and °].

contact	d(H···A)	d(D···A)	<(D−H···A)	symmetry
N(5)-H(5B)...N(1)	2.00	2.660(3)	130.4	*x, y, z*
O(3)-H(3B)...O(2)	1.71	2.576(2)	165.3	*-x+1, y, -z+1/2*
N(3)-H(3A)...O(1)	1.93	2.796(2)	167.2	*x, y, z*
N(4)-H(4A)...O(2)	1.94	2.796(3)	162.7	*x-1/2, -y+3/2, z-1/2*
N(5)-H(5A)...O(1)	2.01	2.885(3)	173.7	*x-1/2, -y+3/2, z-1/2*
N(2)-H(2F)...O(4)	1.99	2.825(2)	157.3	*x, y, z*
N(4)-H(4B)...O(3)	2.08	2.899(2)	153.6	*x, y, z*

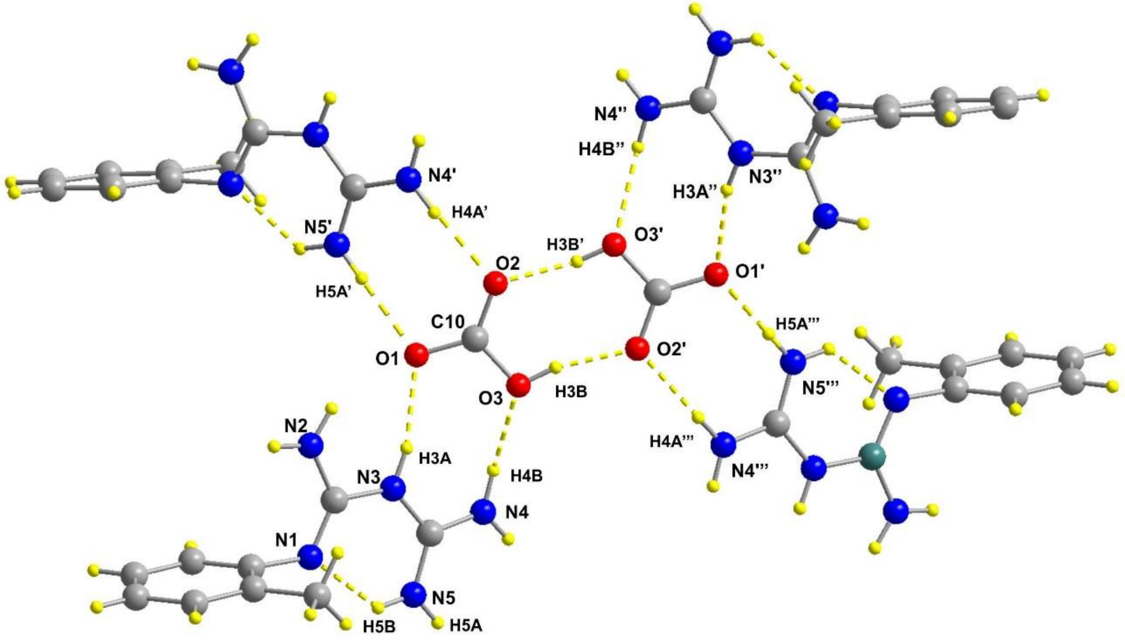

Figure S3. Hydrogen bonding in the structure of compound **3**. Hydrogen bonds are depicted with thick yellow dashed lines.

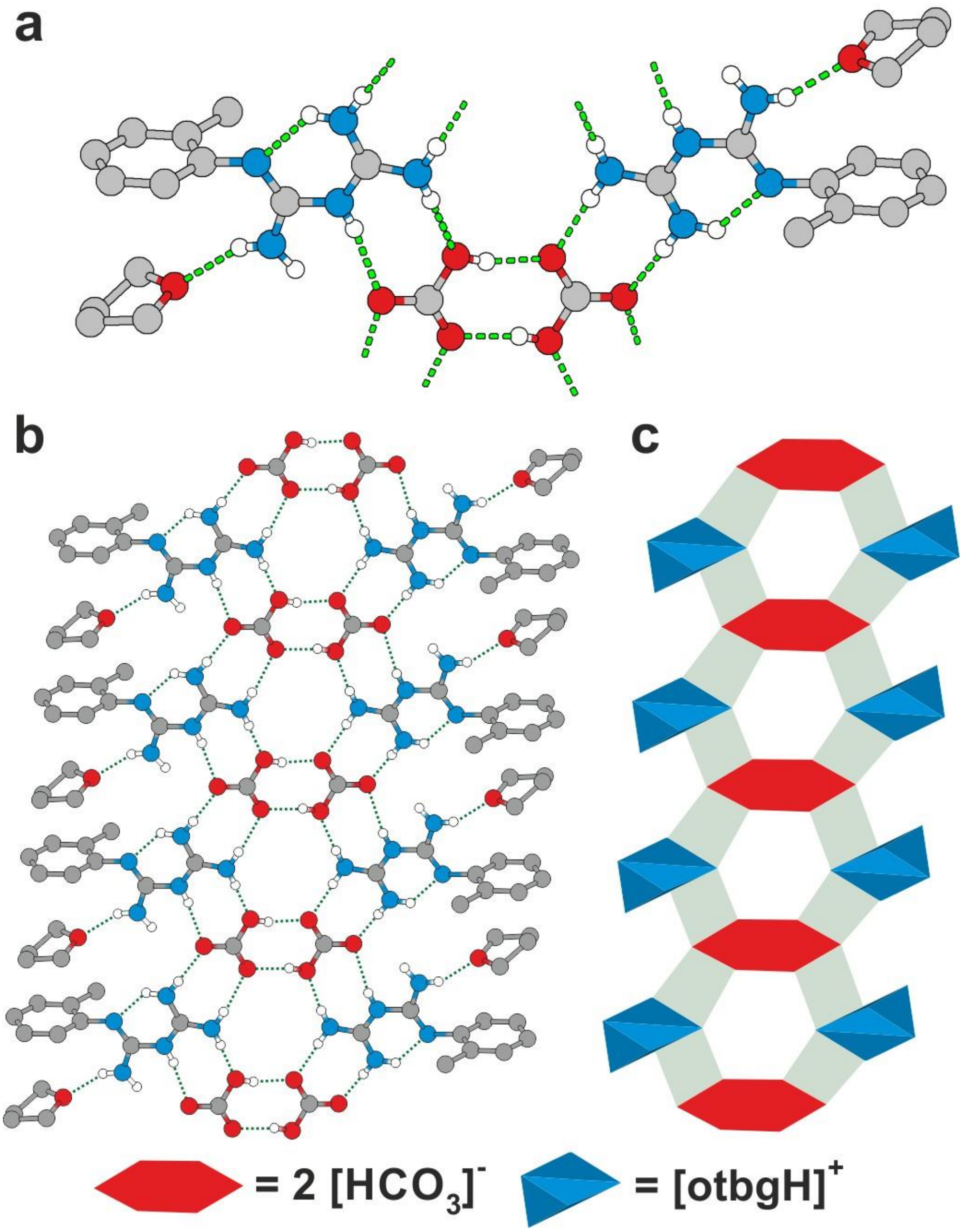

Figure S4. Crystal structure of **3** demonstrating the hydrogen-bonded network structure: a) simple building units, b) supramolecular rosette-ribbon hydrogen-bonded network, c) topological representation of the rosette-ribbon network. C = grey, N = blue, O = red, H = white.

3. Powder X-ray diffraction study

Crystal structure solution of compound 2 by powder X-ray diffraction

PXRD patterns were collected on the PANalytical Empyrean diffractometer equipped with 1D X'Celerator detector. Measurements employed Ni-filtered Cu Kα radiation of a copper sealed tube (40 kV, 40 mA). Samples were placed on the Si zero-background holder and measured in Bragg-Brentano geometry Diffraction patterns were measured in the 2θ range of 5 – 90 degrees with a step size of 0.008 degree.

Crystal structure of compound **2** was solved from powder X-ray diffraction data by direct-space simulated annealing assuming two independent molecular fragments: one for the otbgH moiety and the other for the hydrogencarbonate anion. Structure solution was recognized when a chemically reasonable network of hydrogen bonds was formed with no significant overlap between the fragments and a reasonably good fit to the experimental pattern. Since otbg can be protonated on two sites, which then also changes the proton position at the hydrogencarbonate, we have tested both possibilities in a Rietveld refinement. Since this change in configuration is subtle for X-ray diffraction, the protonation site on otbg could not be unambiguously determined solely from a Rietveld refinement and the two configurations were subjected to quantum-chemical solid-state optimization. Density functional theory (DFT) based calculations were performed by using the Quantum ESPRESSO package. [3–5] Two different setups were used for the present calculations. In the first setup ultrasoft pseudopotentials[6] from Quantum ESPRESSO pseudopotential database [7] were used along with the plane wave cut-off for wave functions of 40.0 Ry and for charge density and potential of 400.0 Ry. Exchange-correlation energy was calculated within generalized gradient approximation (GGA) according to Perdew et al. (PBE).[8,9] To include van der Waals corrections, Grimme DFT-D3 approximation[10] was used. In the second setup GBRV ultrasoft pseudopotentials[11] were employed with plane wave cut-off for wave functions of 65.0 Ry and that for charge density and potential of 650.0 Ry. For calculation of exchange-correlation energy vdW-DF-cx functional was employed.[12,13] In all calculations 4×6×3k-mesh was used for sampling of the Brillouin zone. Optimisation of the second starting configuration resulted in a proton shift leading to a +2 charged otbg cation and a carbonate anion. This configuration was dismissed as being to unlikely and not in agreement with spectroscopic evidence (see main text). Moreover, energies of the two optimised geometries differed by a few kJ/mol favoring the configuration with the hydrogencarbonate.

The optimized configuration was reintroduced into Rietveld refinenement using restraints on bond distances, angles and planarity restraints. The final Rietveld plot is given in Figure S1. All calculation sere performed using the program Topas (version 4.2, Bruker-AXS, Karlsruhe). The cif file, along with the diffraction pattern, is deposited with the Cambridge Crystallographic Data Center under the deposition number CCDC 2093967 and is available upon request.

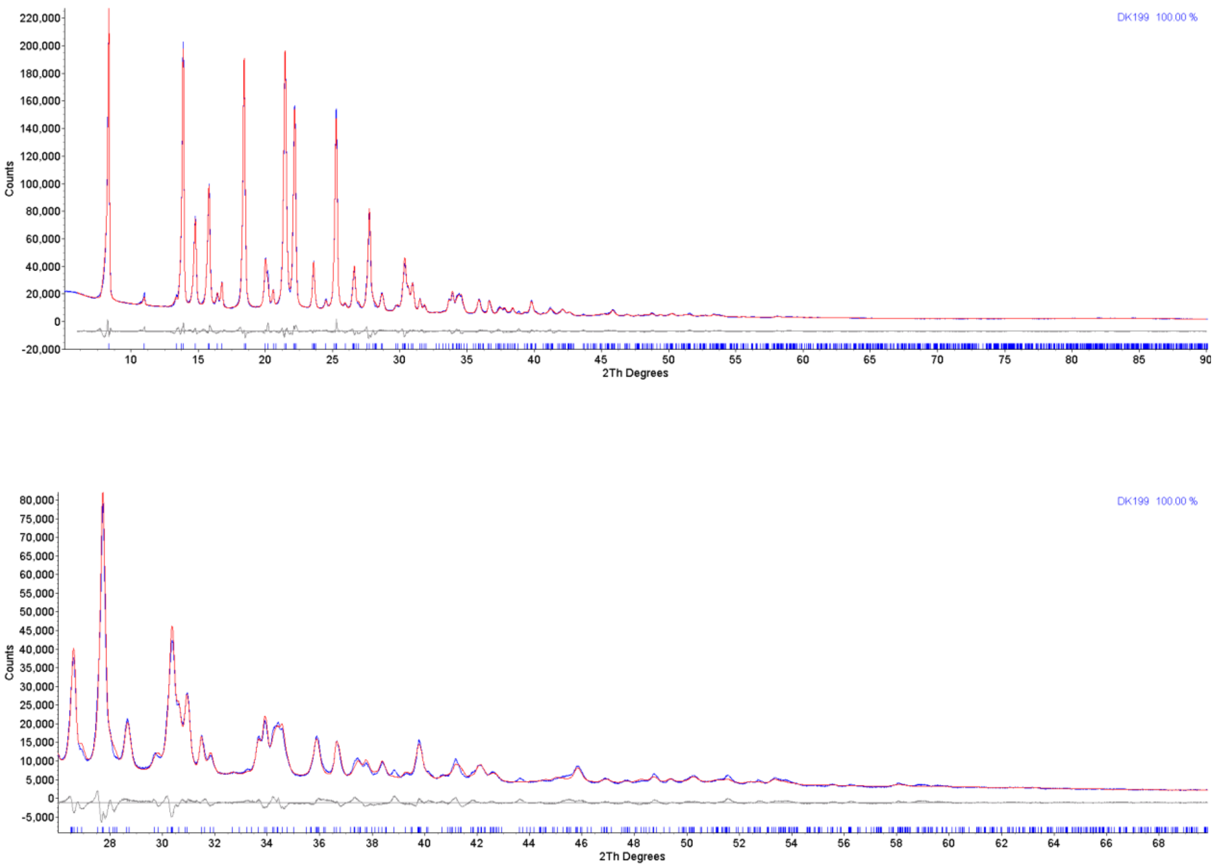

Figure S5. (top)The full Rietveld plot and (bottom) high-angle region for the crystal structure of compound **2**. Color code: blue – measured, red – calculated, grey – difference.

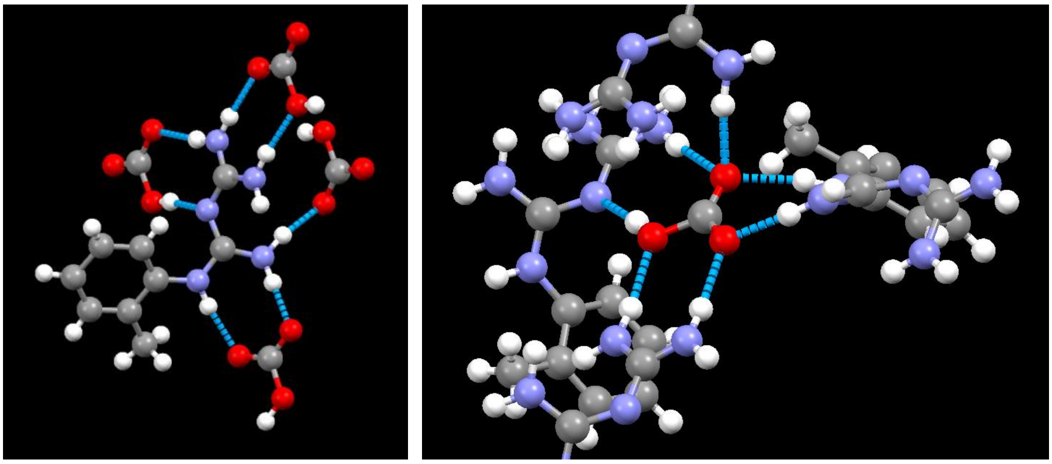

Figure S6. Hydrogen bonding around (left) otbgH and (right) hydrogencarbonate in the structure of compound **2**. Hydrogen bonds are depicted with thick blue dashed lines.

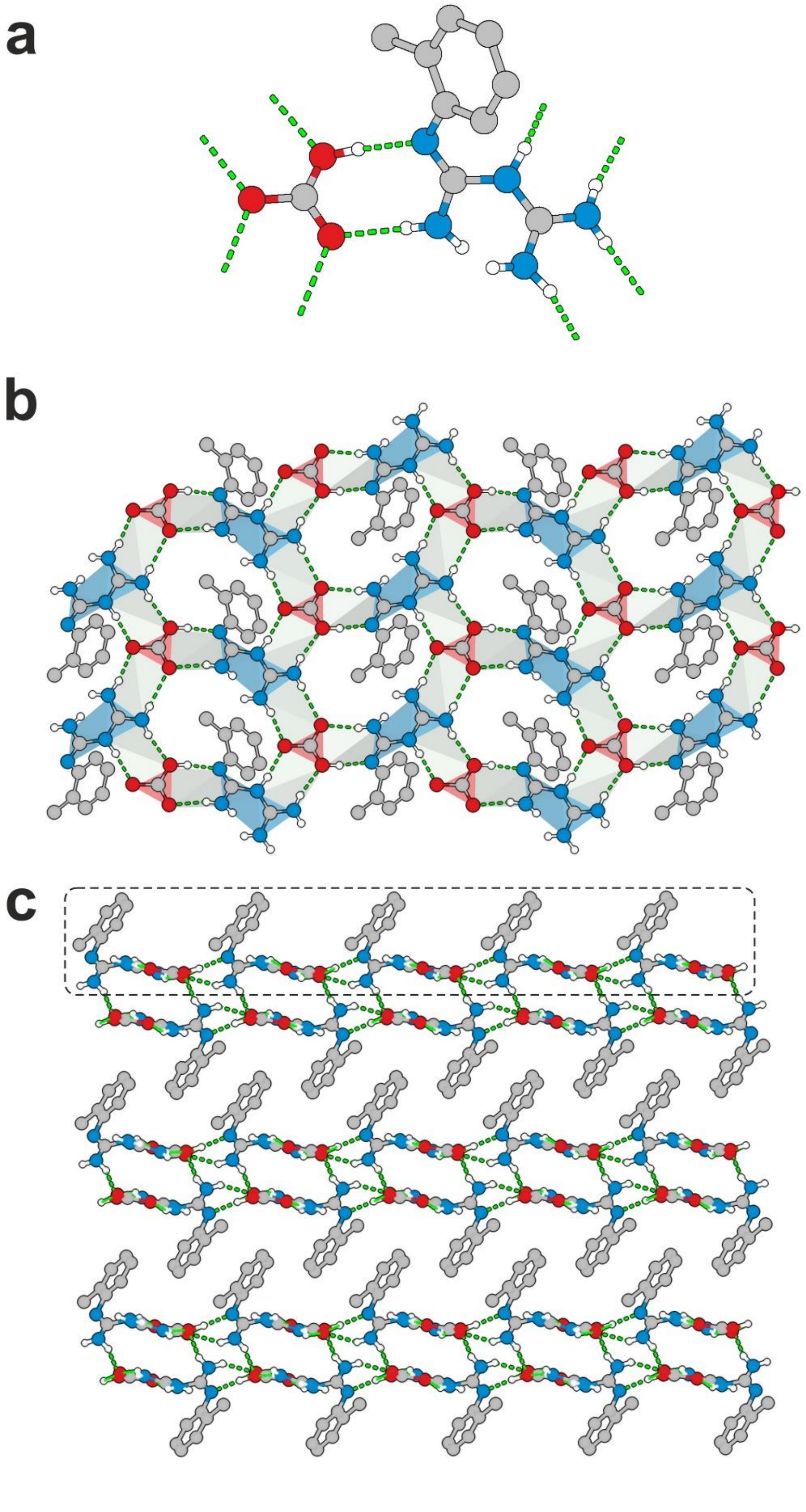

Figure S7. Crystal structure of **2** demonstrating the hydrogen-bonded network structure: a) simple building units, b) 2D supramolecular hydrogen-bonded network (half-layer), c) stacking of the 2D layers (half-layer marked in rectangle),

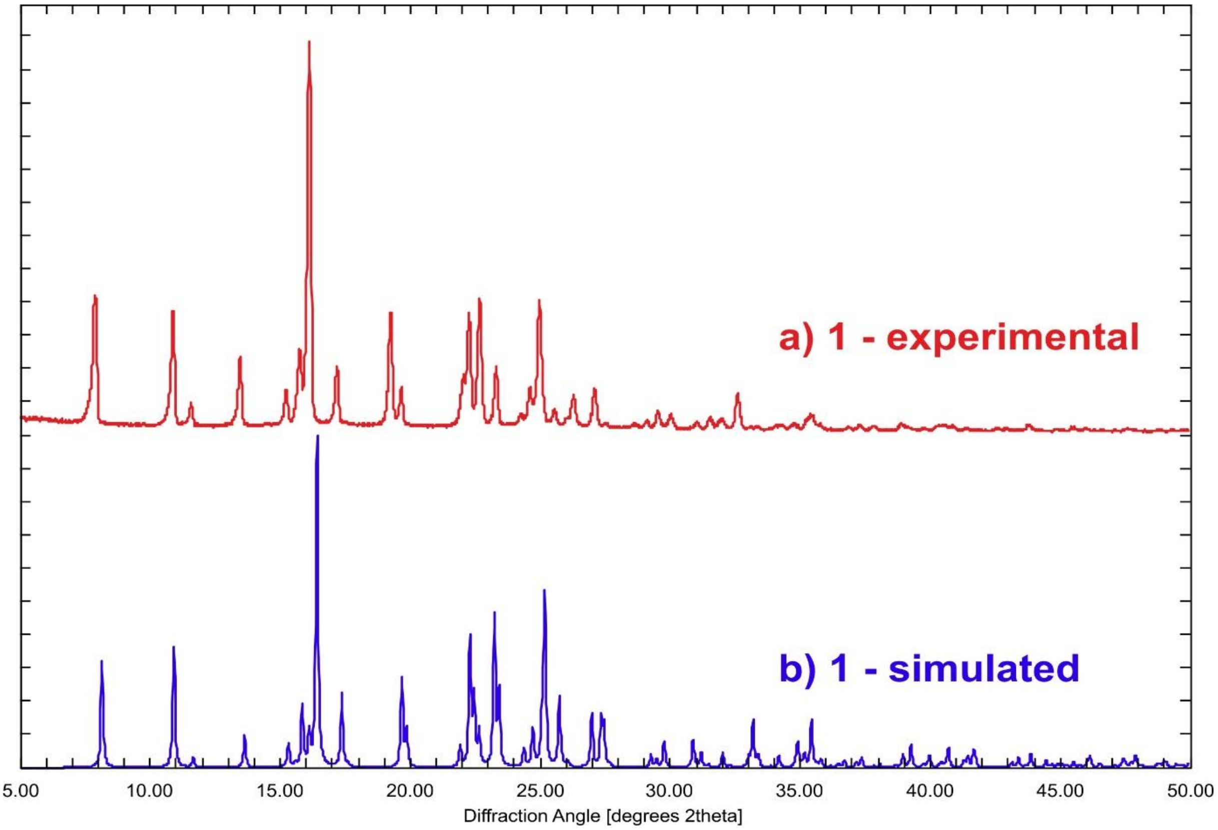

Figure S8. Experimental (a) and simulated (b) diffractograms of compound **1**.

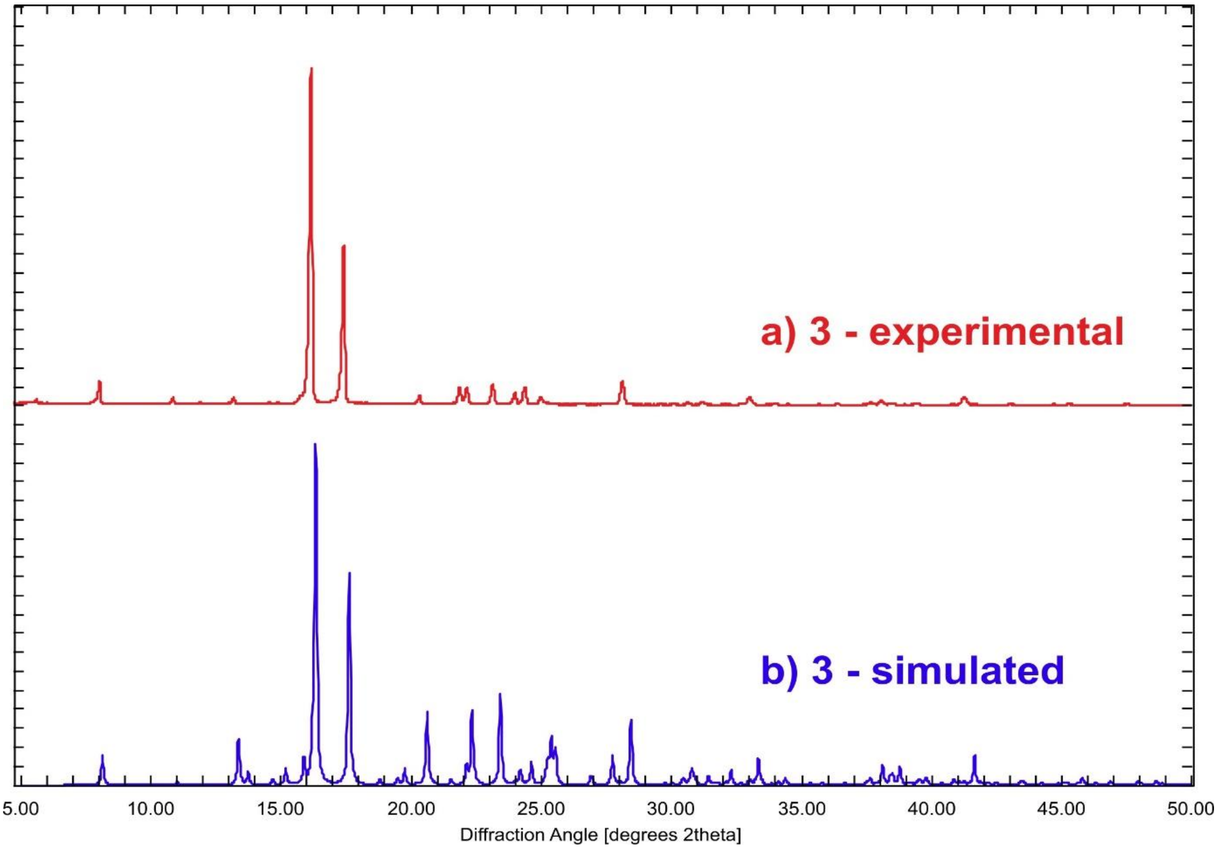

Figure S9. Experimental (a) and simulated (b) diffractograms of compound **3**. The diffractogram simulation involved the preferred orientation effects.

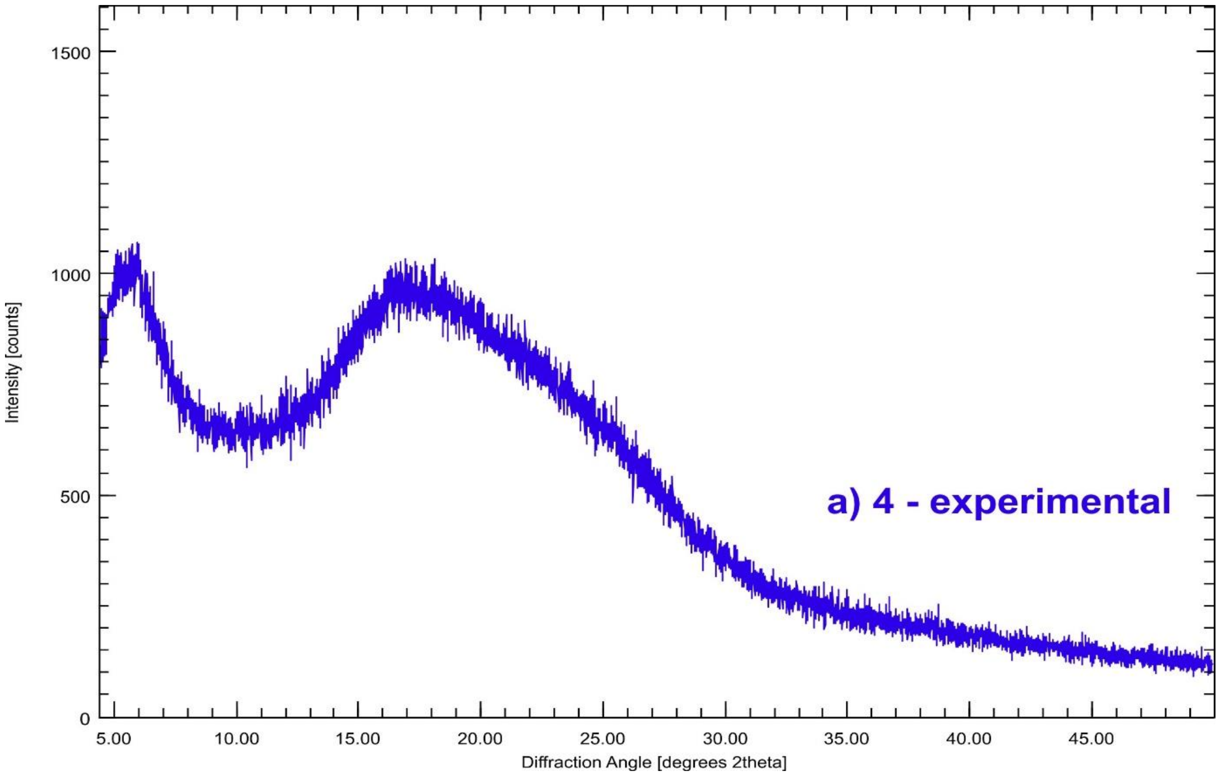

Figure S10. Experimental diffractogram of compound **4**.

4. NMR study

Solid state NMR spectra were recorded using Bruker Avance II 500 MHz spectrometer. Spectra were collected with 3-channel 4 mm CP-MAS probe, spin rate of 10 kHz, using glycine as a reference. Solution NMR spectra were recorded using Varian 400 MHz spectrometer.

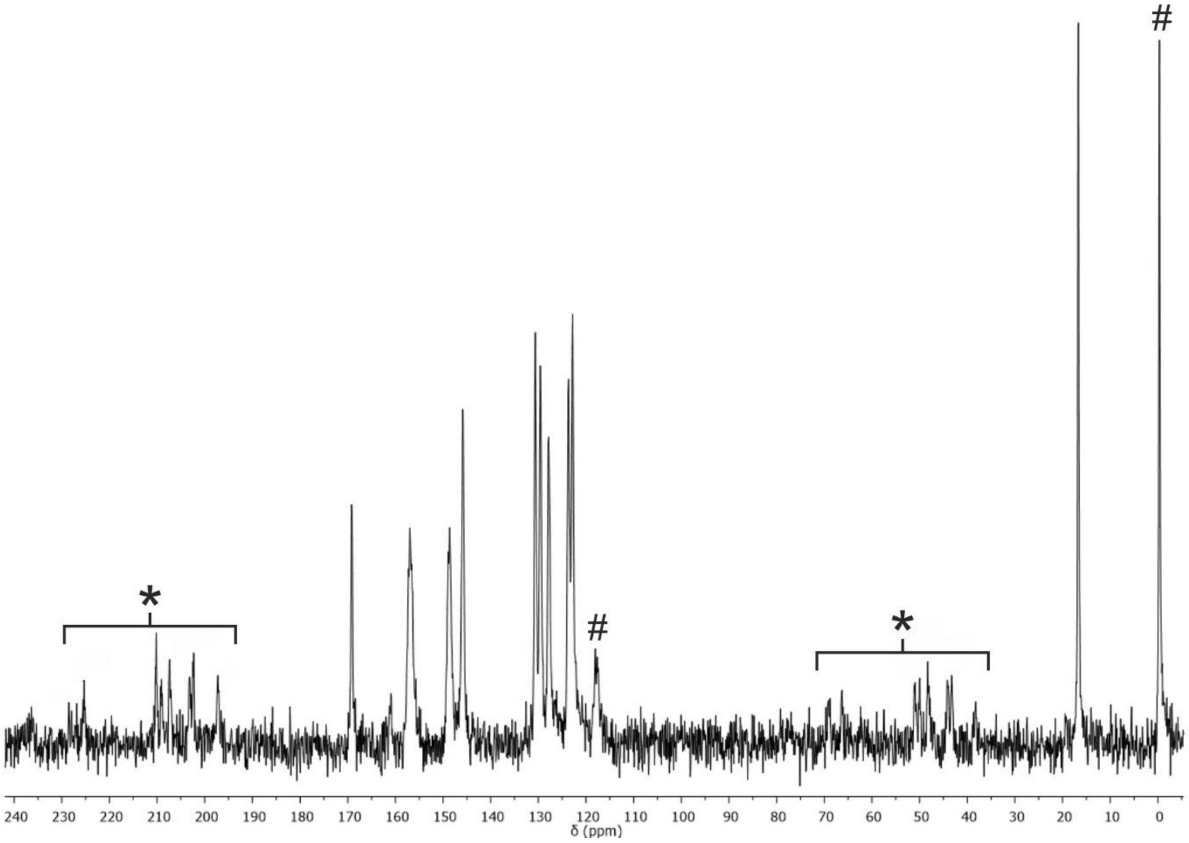

Figure S11. ^{13}C CPMAS NMR spectrum of **1**: δ = 169.16 ($\mathit{C}O_3$), 156.97 ($\mathit{C}N_3$), 148.63 ($\mathit{C}N_3$), 145.88 (N-C_{arom}), 130.66 – 122.80 (C_{arom}), 16.69 ($\mathit{C}H_3$). # = solvent signals (MeCN), * = rotational artifacts.

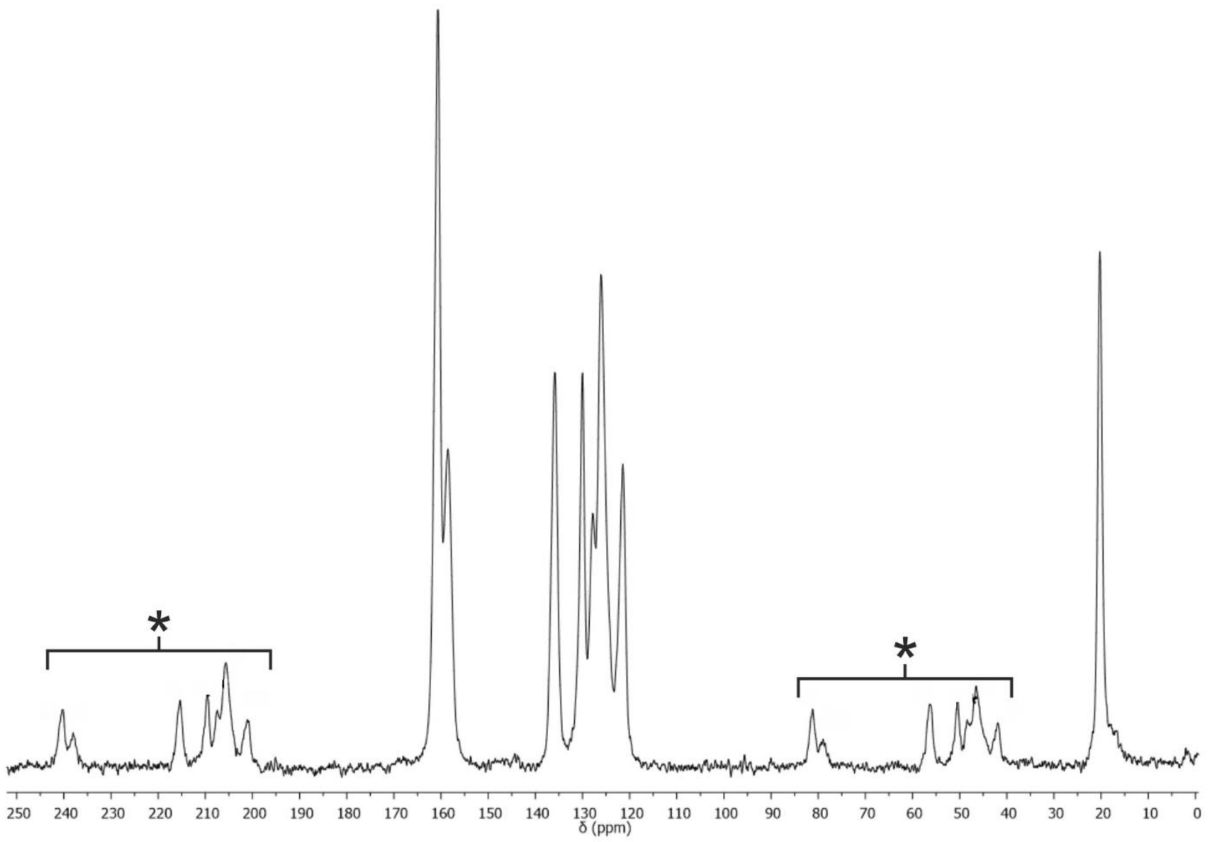

Figure S12. ^{13}C CPMAS NMR spectrum of **2**: δ = 160.72 (H*C*O_3), 158.50 (*C*N_3), 135.87 (*C*N_3), 130.02 – 121.45 (C_{arom}), 20.28 (*C*H_3). * = rotational artifacts.

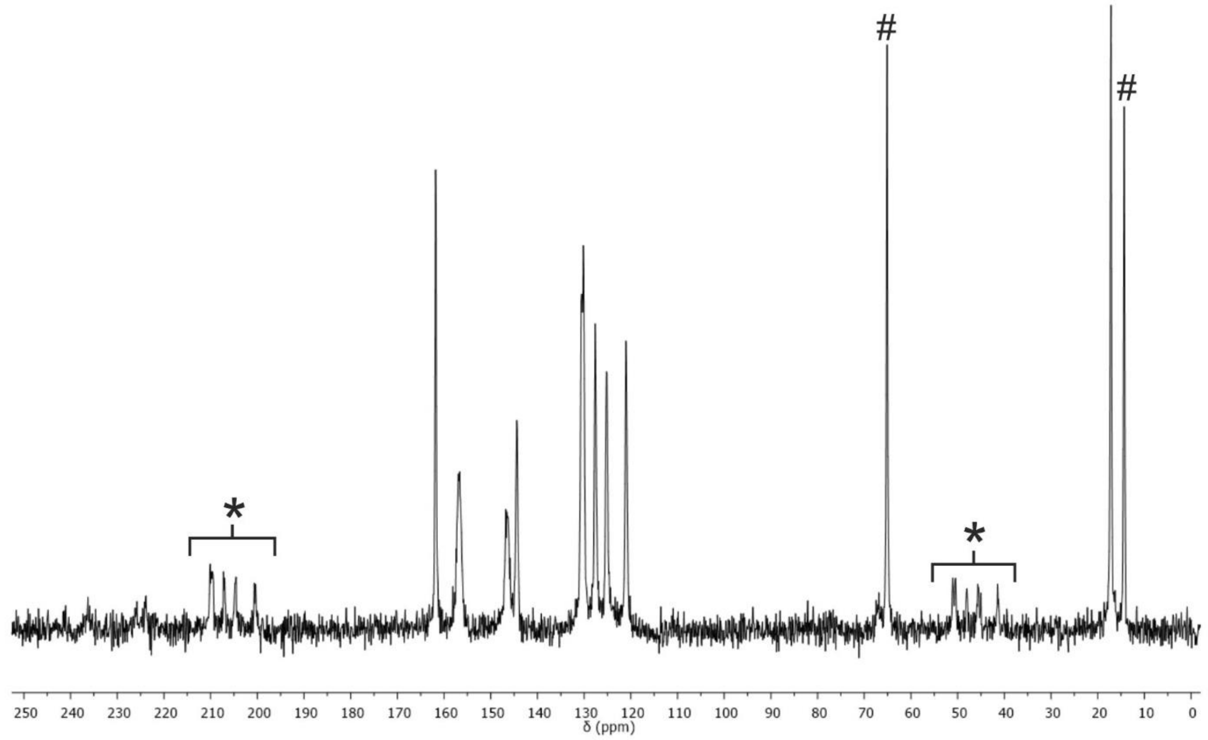

Figure S13. ^{13}C CPMAS NMR spectrum of **3**: δ = 161.82 (H*C*O_3), 156.93 (*C*N_3), 146.78 (*C*N_3), 144.39 (N-C_{arom}), 130.53 – 121.03 (C_{arom}), 17.17 (*C*H_3). # = solvent signals (THF), * = rotational artifacts.

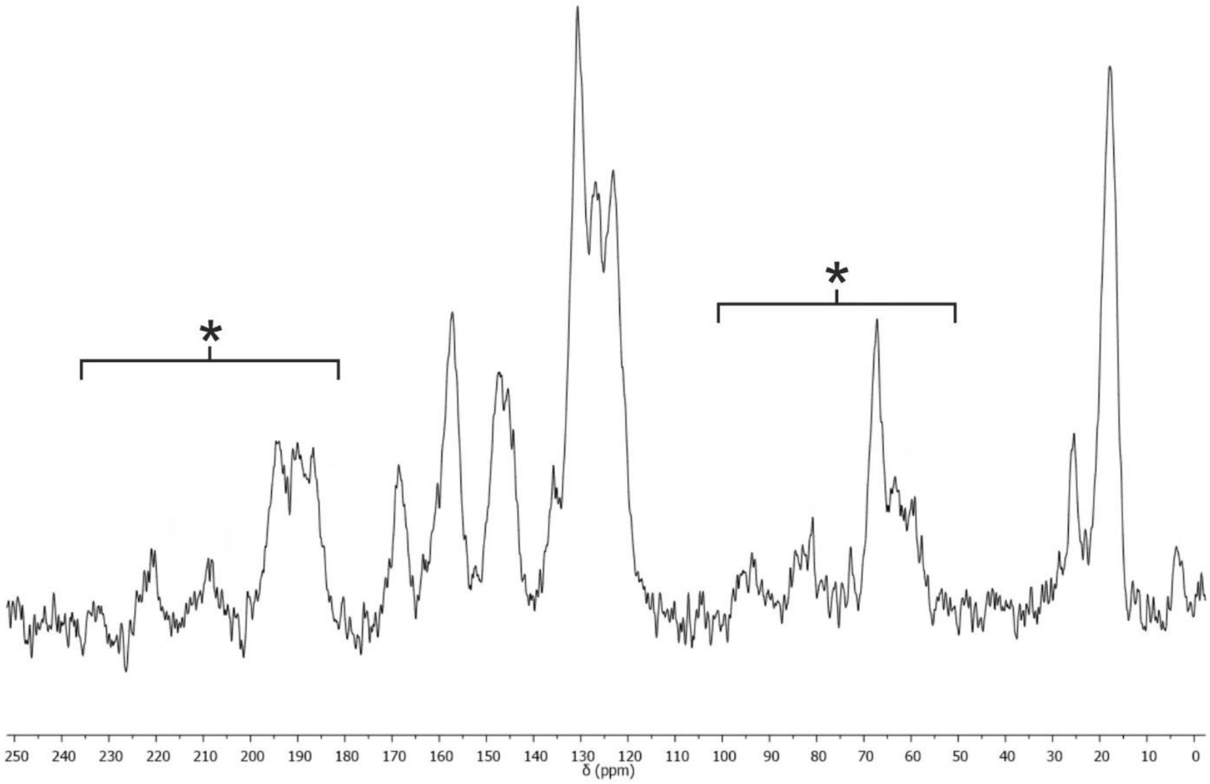

Figure S14. ^{13}C CPMAS NMR spectrum of **4**. δ = 168.51 (CO_3), 157.33 (CN_3), 147.22 (CN_3), 136 – 123 (C_{arom}), 17.99 (CH_3). * = rotational artifacts.

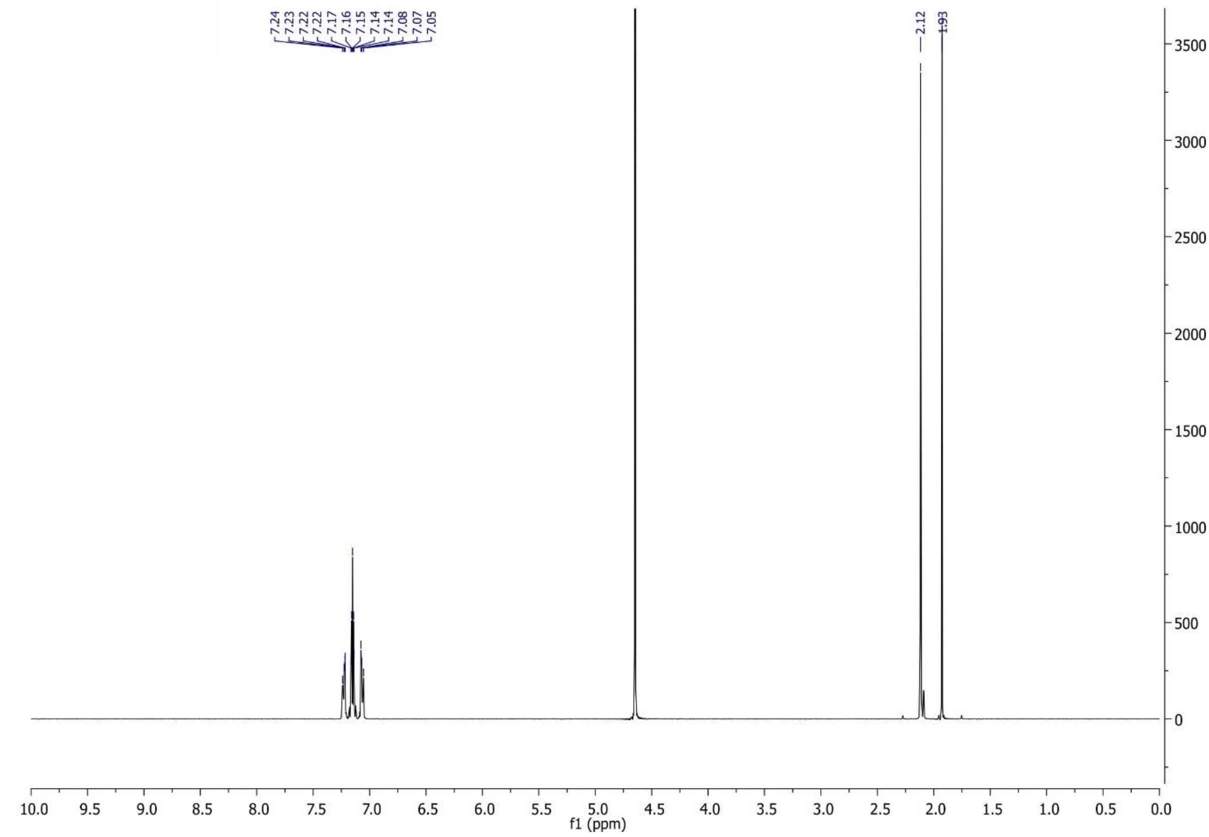

Figure S15. ^1H NMR spectrum of **1** in D_2O.

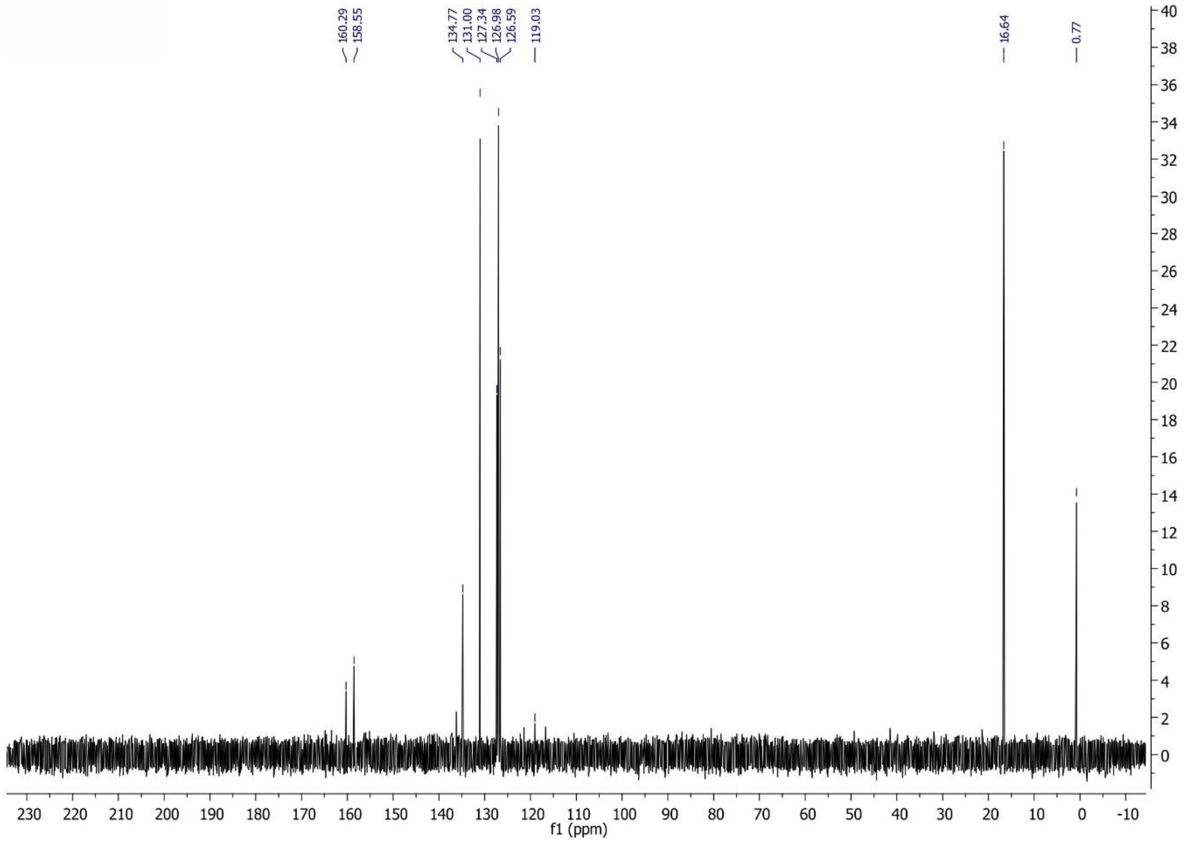

Figure S16. ^{13}C NMR spectrum **1** in D_2O.

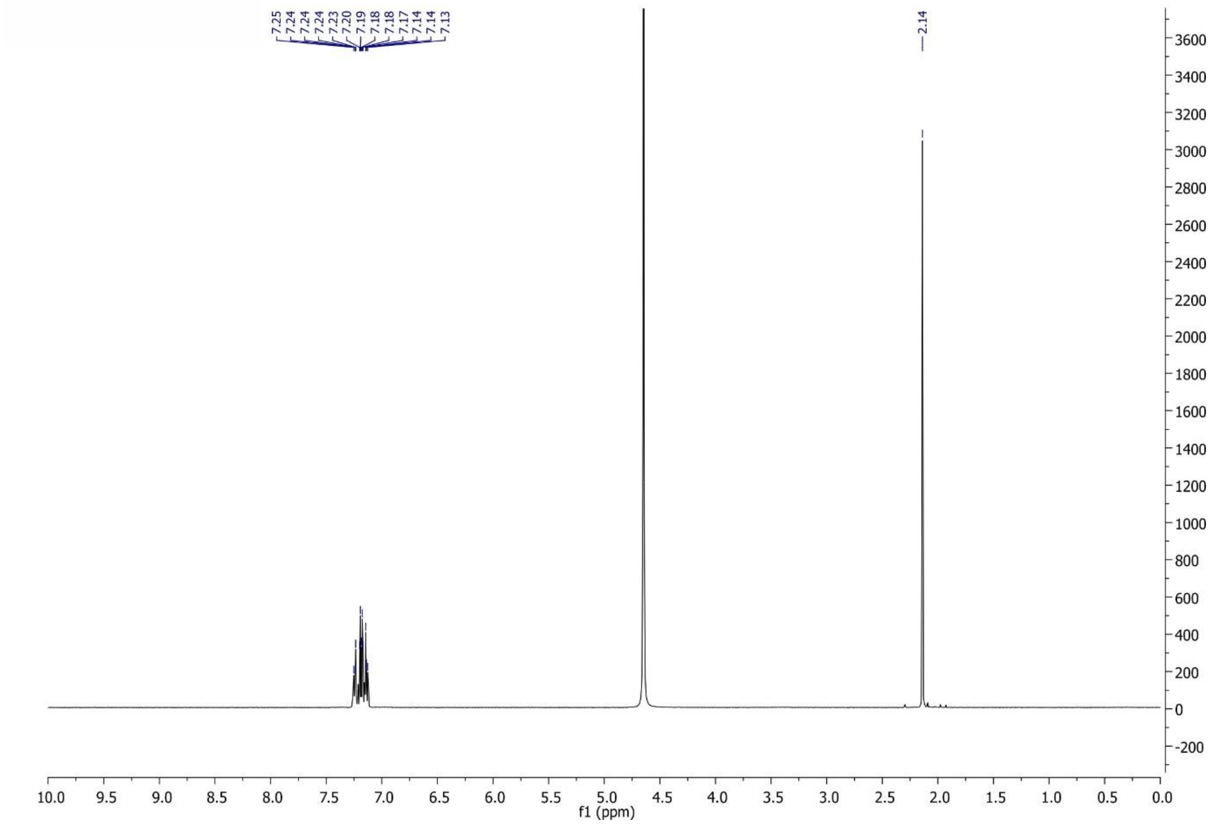

Figure S17. ^{1}H NMR spectrum of **2** in D_2O.

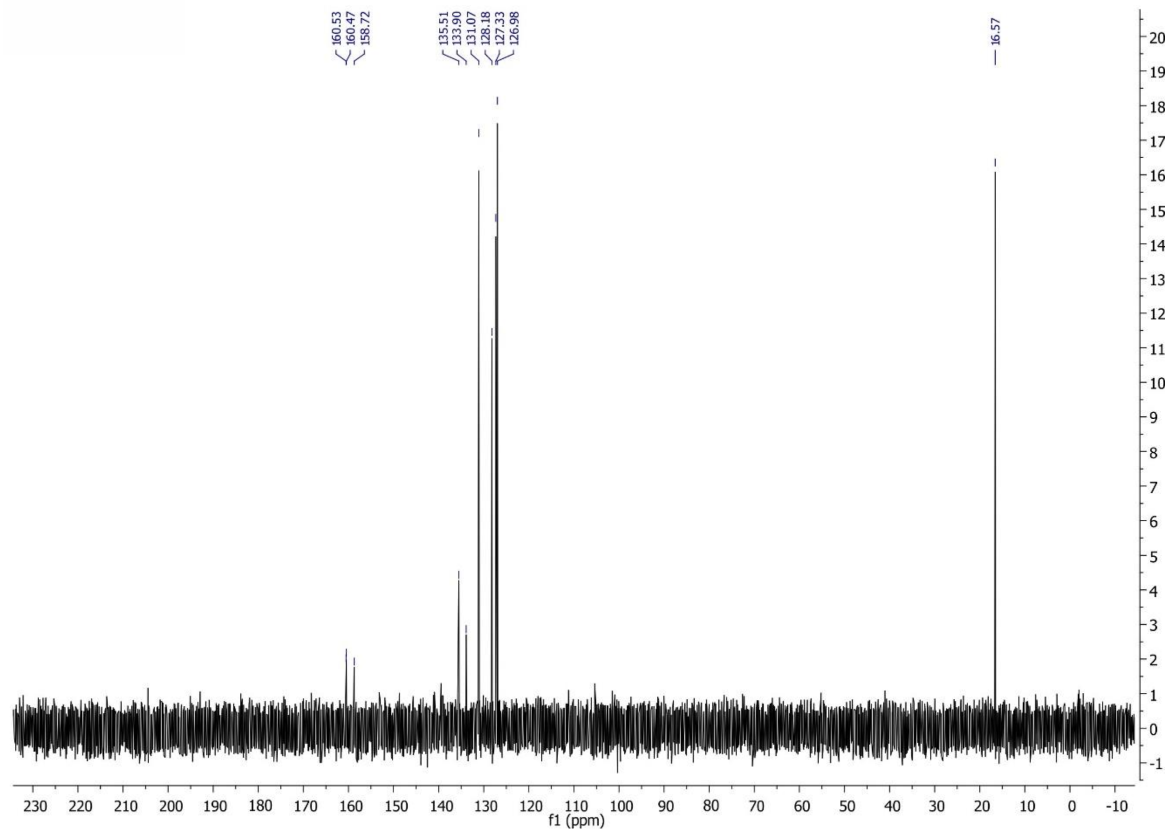

Figure S18. ^{13}C NMR spectrum **2** in D_2O.

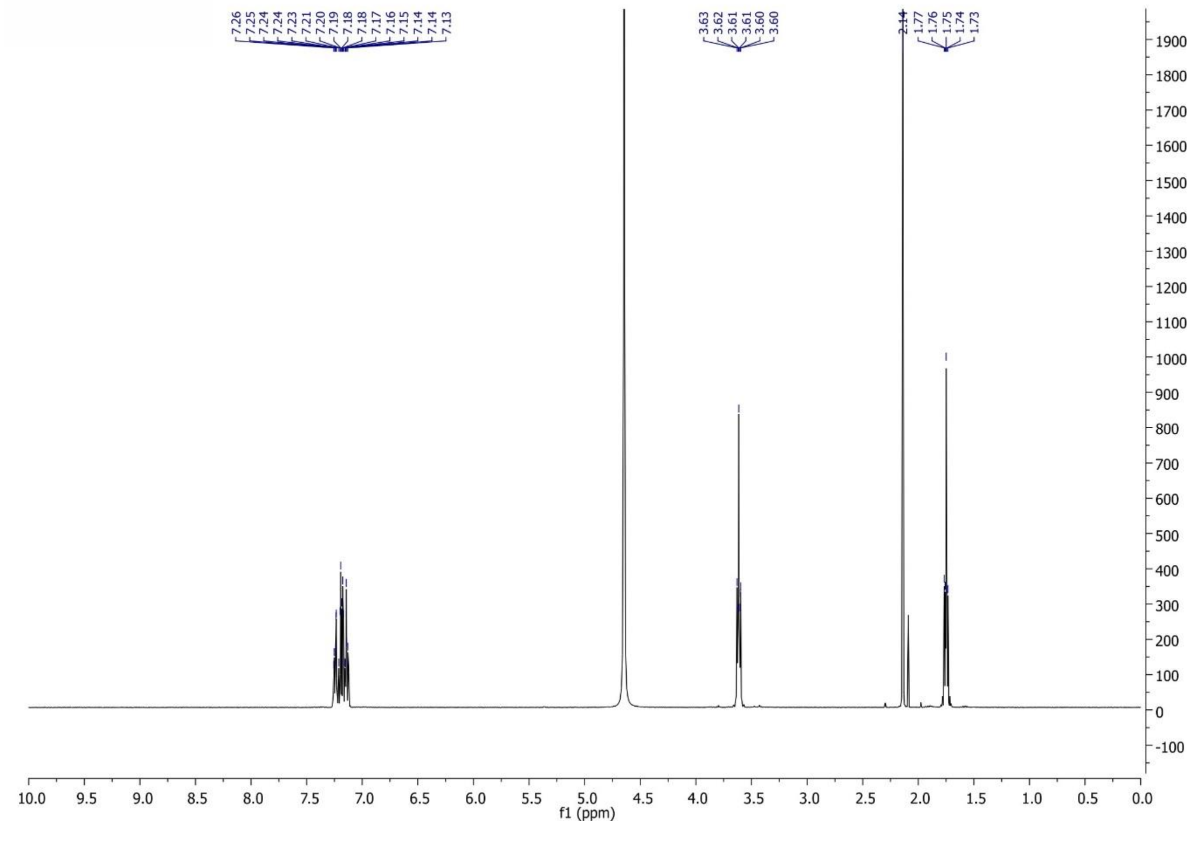

Figure S19. ^{1}H NMR spectrum of **3** in D_2O.

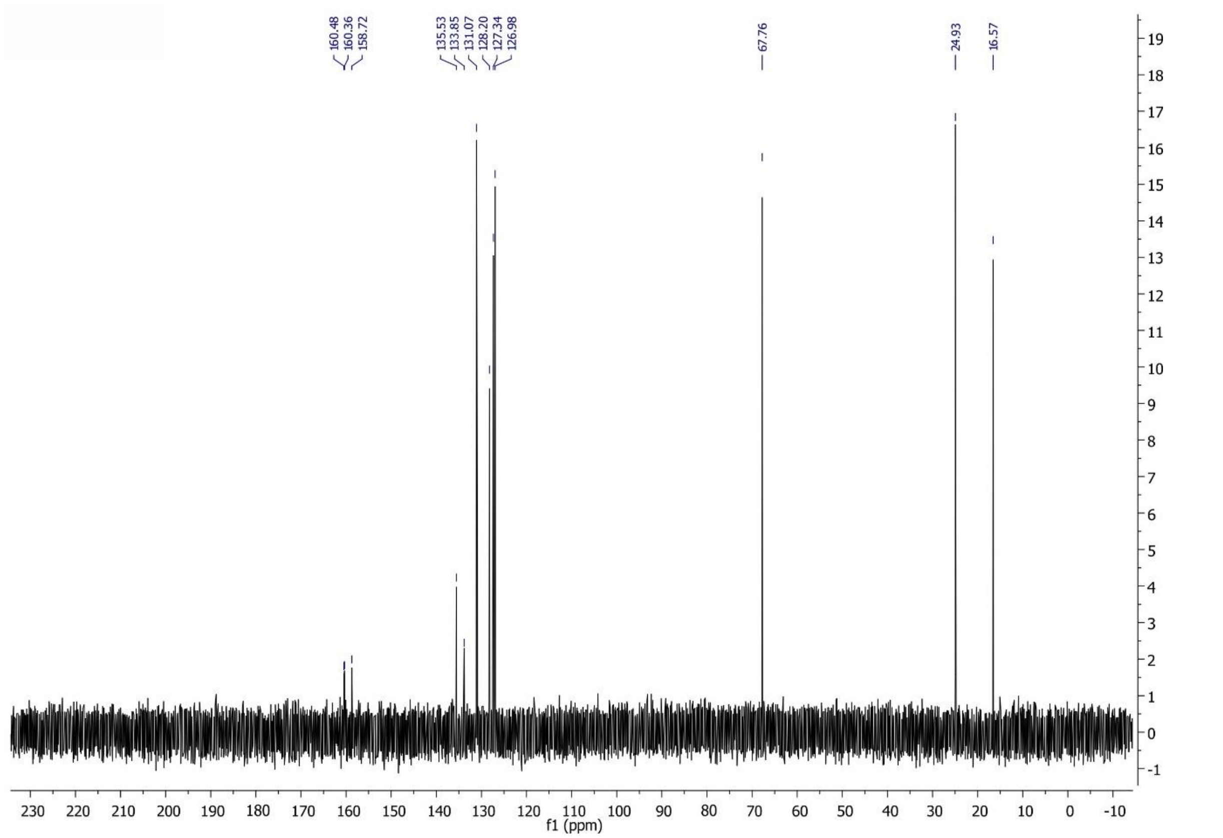

Figure S20. ^{13}C NMR spectrum **3** in D_2O.

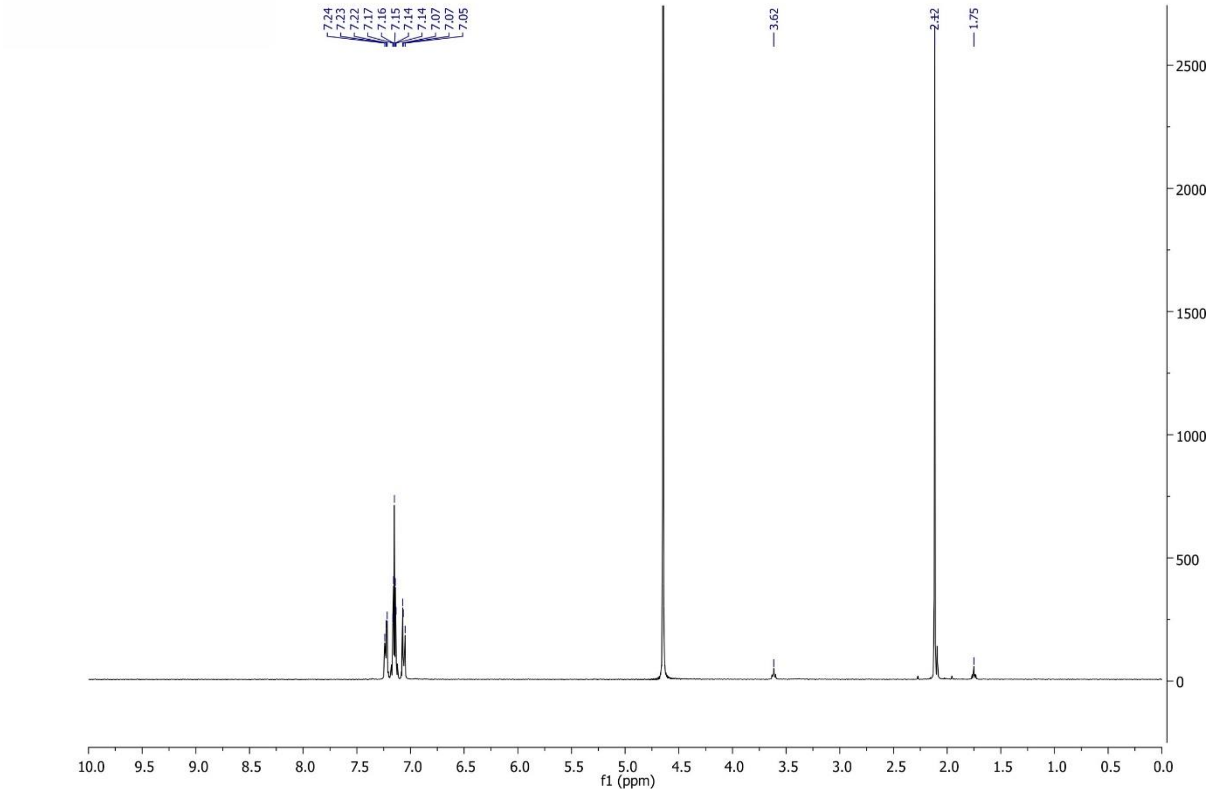

Figure S21. ^{1}H NMR spectrum of **4** in D_2O. Traces of THF are visible at 3.62 and 1.75 ppm.

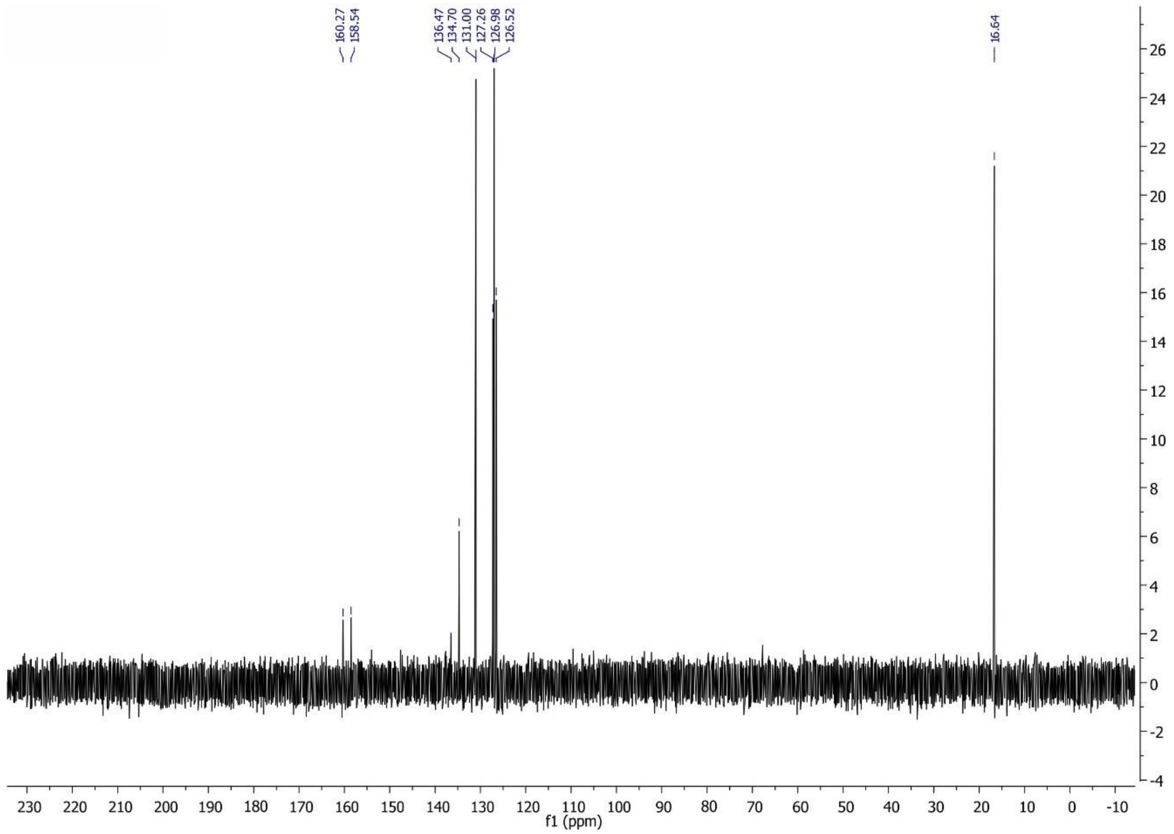

Figure S22. ^{13}C NMR spectrum **4** in D_2O.

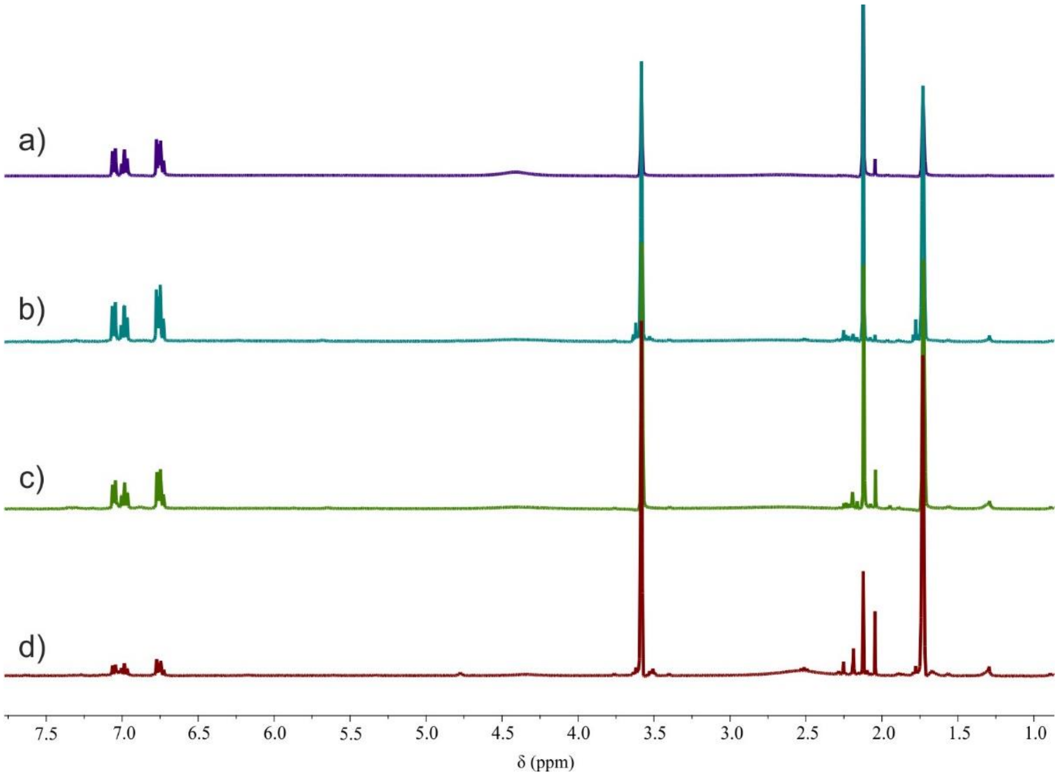

Figure S23. 1H NMR spectra of: a) pure otbg reference, b) thermal decomposition of **3** – product collected after 2h in 100 °C, c)) thermal decomposition of **1** – product collected after 5 cycles of CO2 absorption and release (2h in 100 °C), d) thermal decomposition of **3** – product collected after 5 cycles of CO2 absorption and release (2h in 100 °C), all samples were dissolved in THF-*d8* prior to analysis.

5. Thermogravimetric analysis

Thermogravimetric analysis (TGA) was carried out using a TA Instruments Q600 under a flow of artificial air, to max 400 °C, at a heating rate of 5 °C·min^{-1} (flow rate of 100 mL/min). Open alumina crucibles (5 mm diameter) were used.

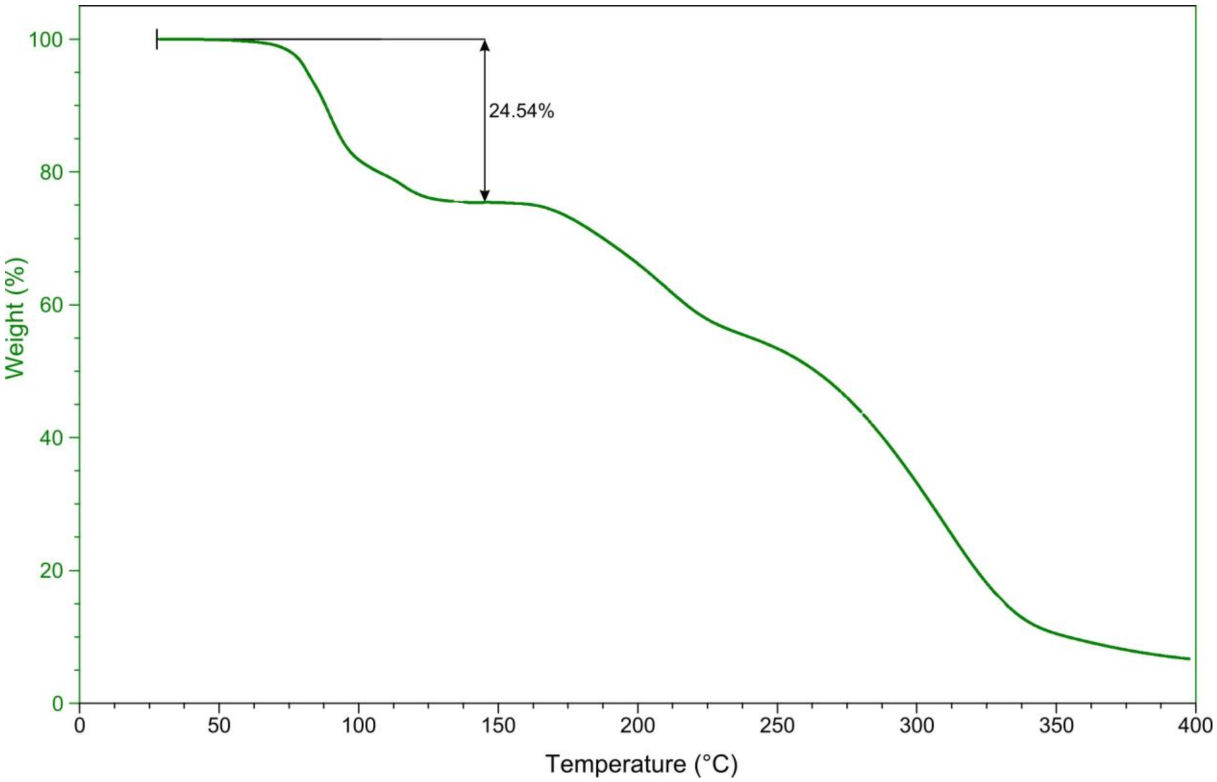

Figure S24. Thermogravimetric analysis of **1**. Theoretical transformation from $[(otbgH)_2(CO_3)(MeCN)_2]$ (**1**) to otbg involves weight loss of 26.97 %, which is close to the measured value (24.54 %).

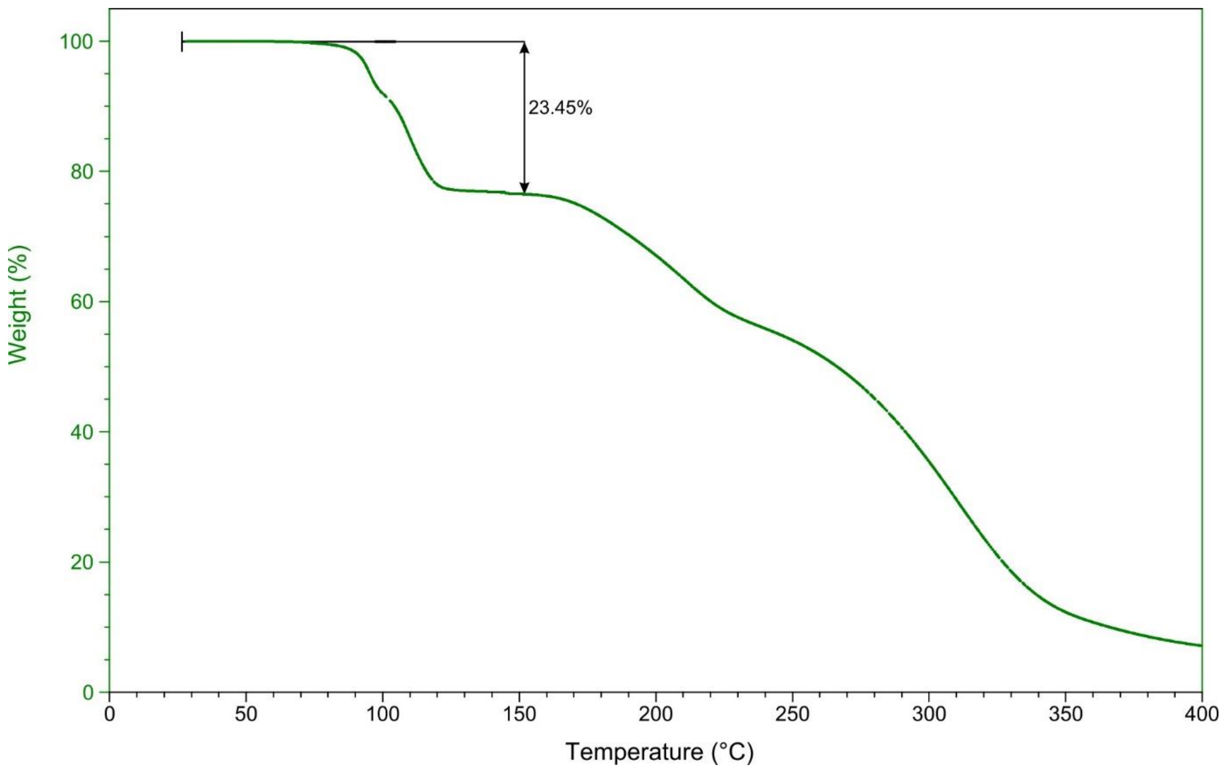

Figure S25. Thermogravimetric analysis of **2**. Theoretical transformation from [(otbgH)(HCO_3)] (**2**) to otbg involves weight loss of 24.09 %, which is close to the measured value (23.45 %).

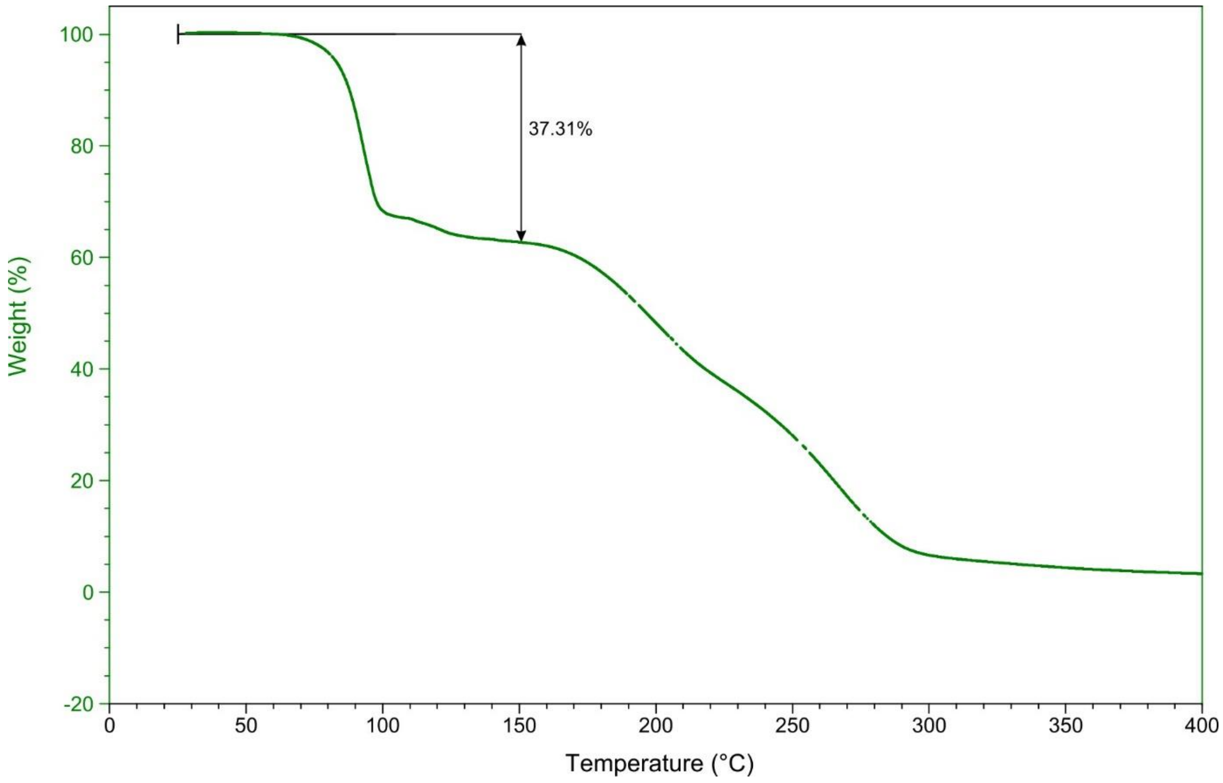

Figure S26. Thermogravimetric analysis of **3**. Theoretical transformation from [(otbgH)(HCO_3)(THF)] (**3**) to otbg involves weight loss of 40.92 %, which is close to the measured value (37.31 %).

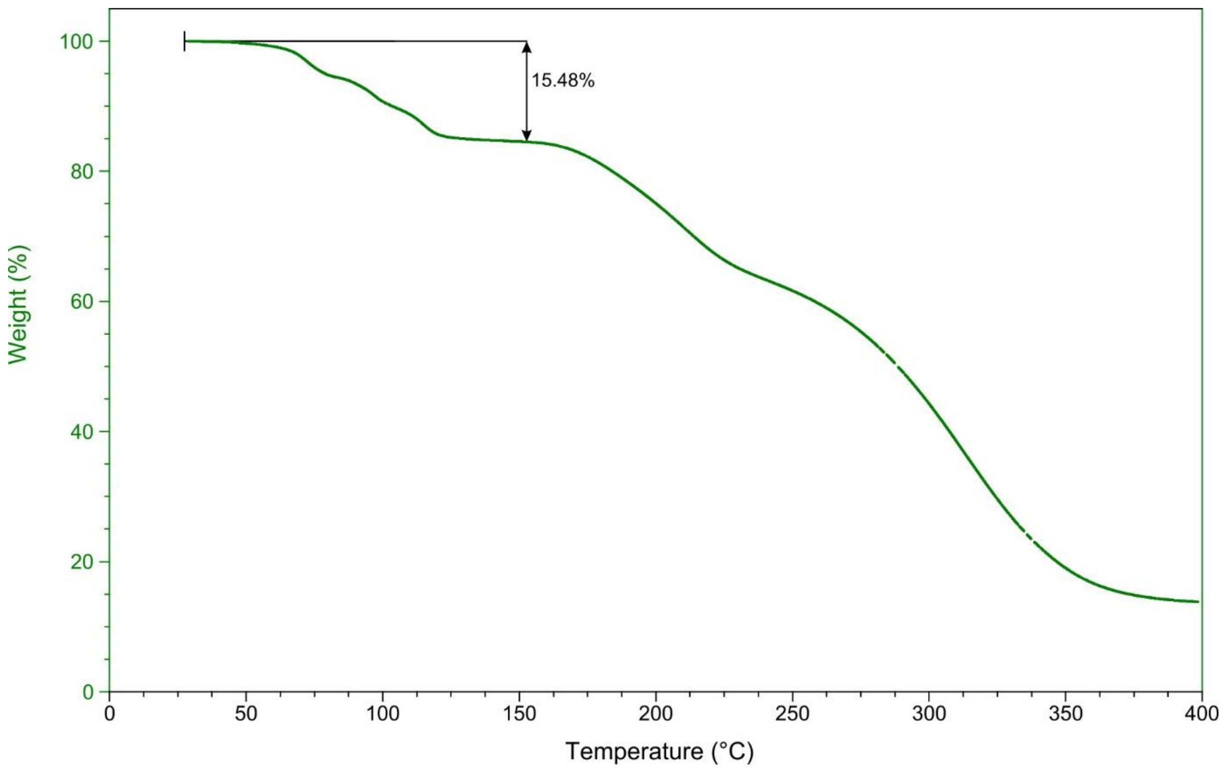

Figure S27. Thermogravimetric analysis of **4**. Theoretical transformation from [(otbgH)$_2$(CO$_3$)] (**2**) to otbg involves weight loss of 13.50 %, which is close to the measured value (15.48 %).

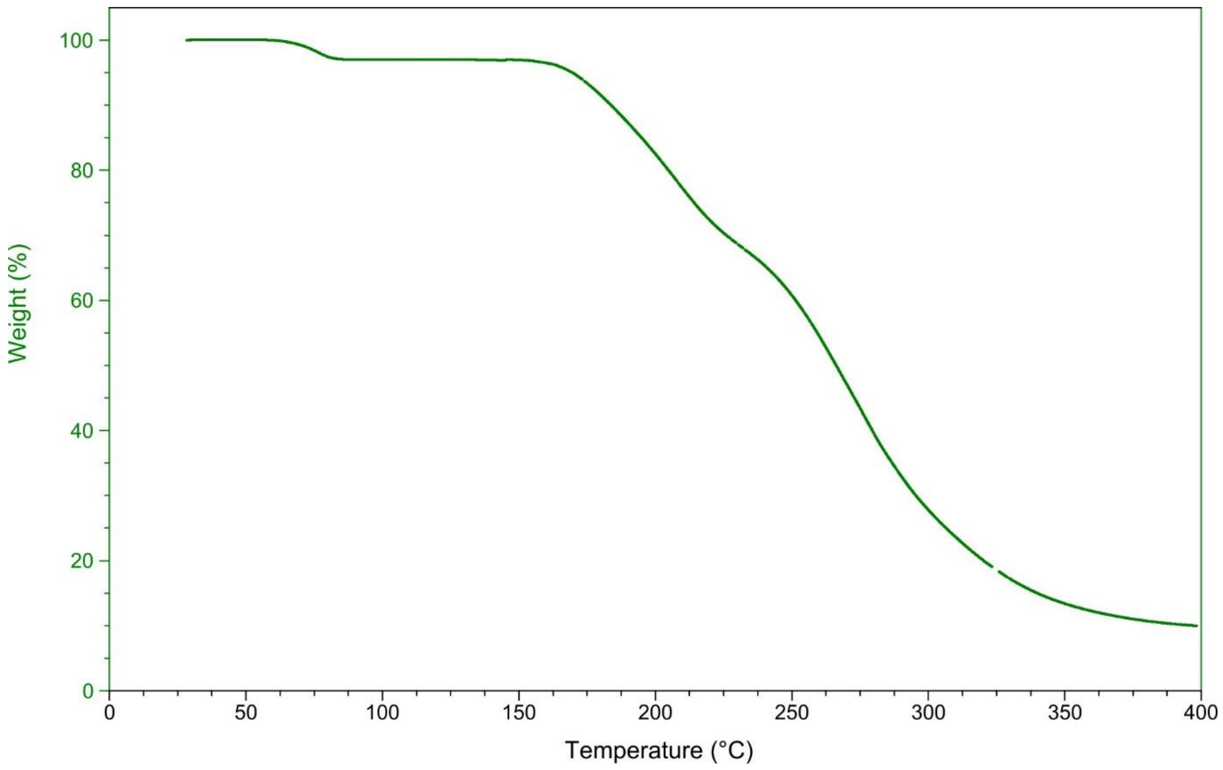

Figure S28. Thermogravimetric analysis of otbg reference.

6. IR spectroscopy

FTIR spectra of powder samples (*ca.* 5 mg) were recorded using the FTIR Bruker-Tensor II System in the ATR mode. Data was processed using the Bruker OPUS software.

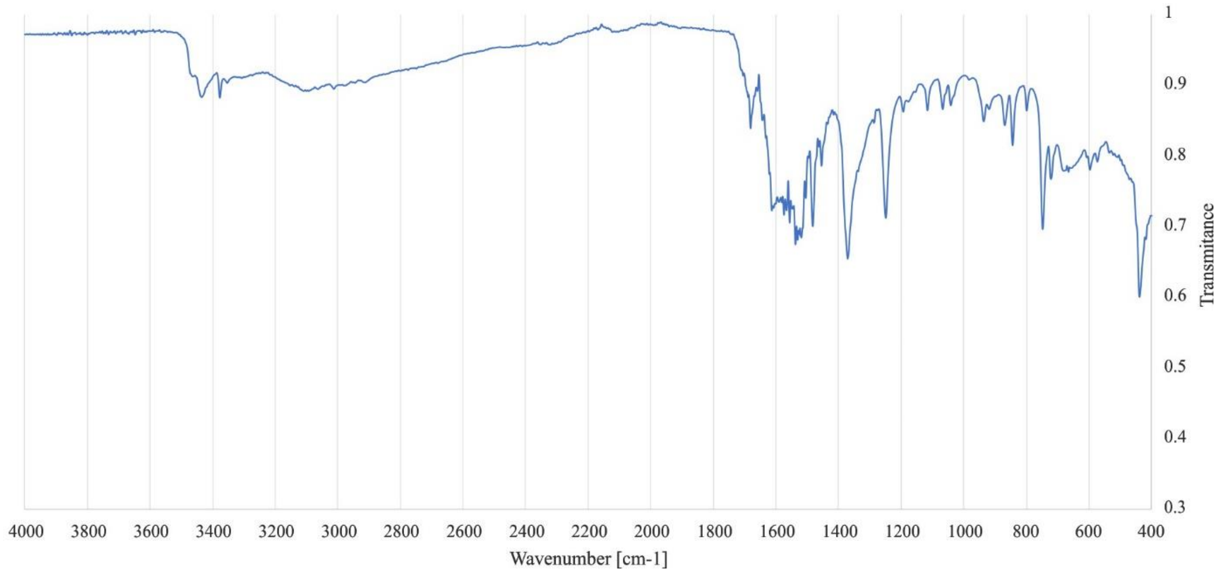

Figure S29. FTIR spectrum of **1**.

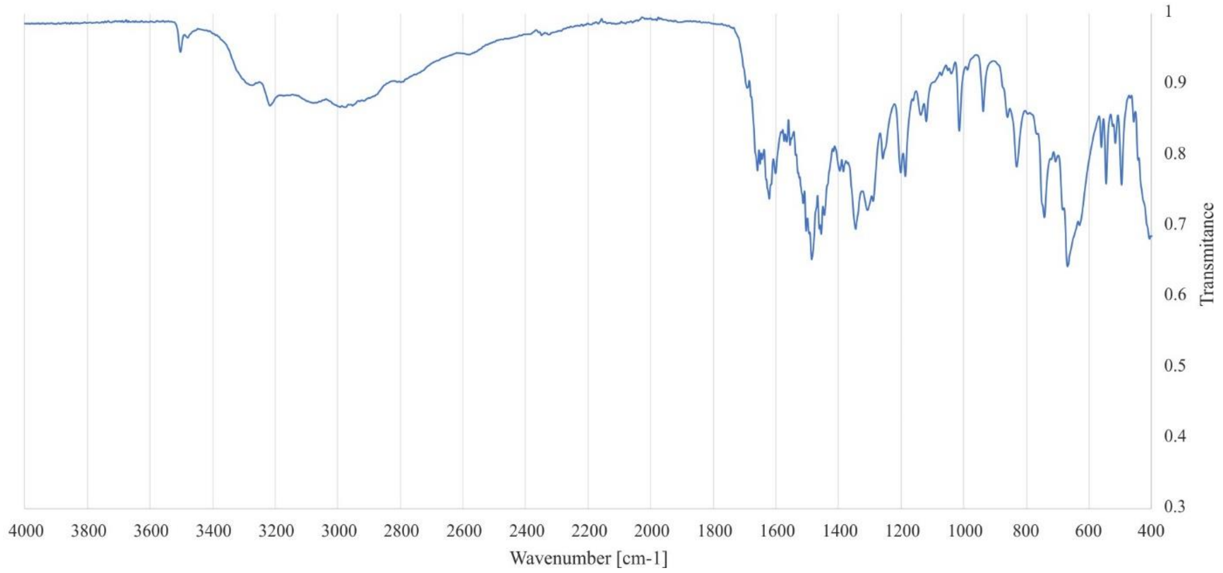

Figure S30. FTIR spectrum of **2**.

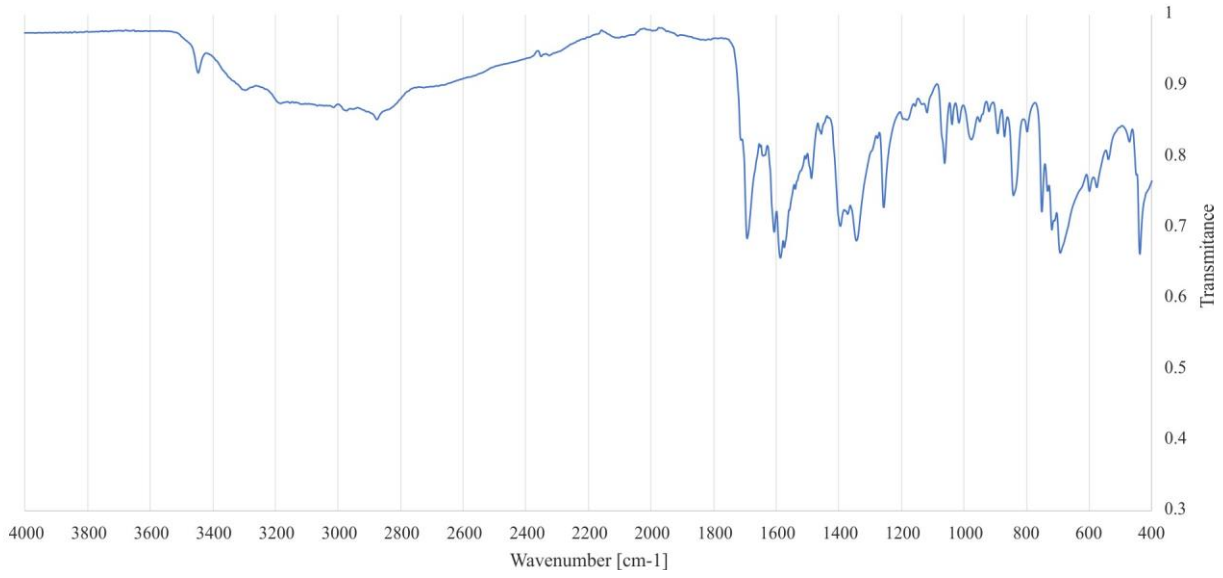

Figure S31. FTIR spectrum of **3**.

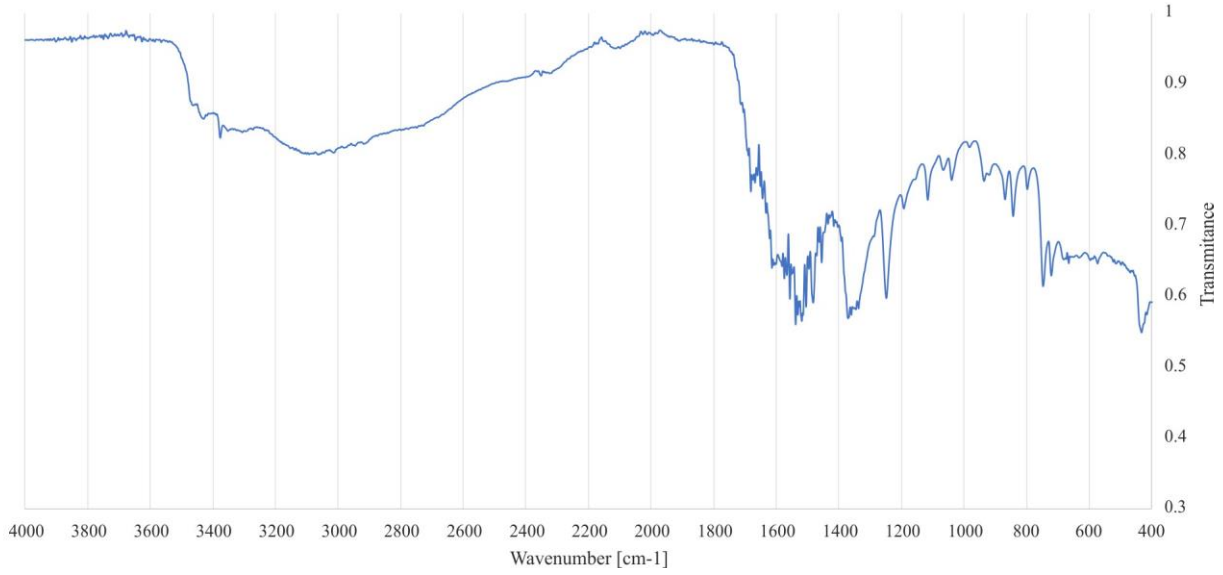

Figure S32. FTIR spectrum of **4**.

7. References

[1] Agilent Technologies, *CrysAlisPro*, Version 1.171.35.21b

[2] G. M. Sheldrick, *Acta Crystallogr. Sect. A* **2008**, *64*, 112–122.

[3] P. Giannozzi, S. Baroni, N. Bonini, M. Calandra, R. Car, C. Cavazzoni, D. Ceresoli, G. L. Chiarotti, M. Cococcioni, I. Dabo, et al., *J. Phys. Condens. Matter* **2009**, *21*, 395502.

[4] P. Giannozzi, O. Andreussi, T. Brumme, O. Bunau, M. Buongiorno Nardelli, M. Calandra, R. Car, C. Cavazzoni, D. Ceresoli, M. Cococcioni, et al., *J. Phys. Condens. Matter* **2017**, *29*, 465901.

[5] P. Giannozzi, O. Baseggio, P. Bonfà, D. Brunato, R. Car, I. Carnimeo, C. Cavazzoni, S. de Gironcoli, P. Delugas, F. Ferrari Ruffino, et al., *J. Chem. Phys.* **2020**, *152*, 154105.

[6] D. Vanderbilt, *Phys. Rev. B* **1990**, *41*, 7892–7895.

[7] We used the pseudopotentials H.pbe-rrkjus.UPF, C.pbe-rrkjus.UPF, O.pbe-rrkjus.UPF and N.pberrkjus.UPF from the Quantum ESPRESSO pseudopotential data base: http://www.quantum-espresso.org/pseudopotentials.

[8] J. P. Perdew, K. Burke, M. Ernzerhof, *Phys. Rev. Lett.* **1996**, *77*, 3865–3868.

[9] J. P. Perdew, K. Burke, M. Ernzerhof, *Phys. Rev. Lett.* **1997**, *78*, 1396–1396.

[10] S. Grimme, J. Antony, S. Ehrlich, H. Krieg, *J. Chem. Phys.* **2010**, *132*, 154104.

[11] K. F. Garrity, J. W. Bennett, K. M. Rabe, D. Vanderbilt, *Comput. Mater. Sci.* **2014**, *81*, 446–452.

[12] K. Berland, C. A. Arter, V. R. Cooper, K. Lee, B. I. Lundqvist, E. Schröder, T. Thonhauser, P. Hyldgaard, *J. Chem. Phys.* **2014**, *140*, 18A539.

[13] K. Berland, P. Hyldgaard, *Phys. Rev. B* **2014**, *89*, 035412.
